# Macrophage ferritin heavy chain/**α**-synuclein regulatory axis modulates ferroptosis during kidney injury

**DOI:** 10.1172/jci.insight.196521

**Published:** 2026-03-10

**Authors:** Tanima Chatterjee, Sarah Machado, Kellen Cowen, Mary E. Miller, Bronte Johnson, Yanfeng Zhang, Laura A. Volpicelli-Daley, Lauren A. Fielding, Rudradip Pattanayak, Frida Rosenblum, László Potor, György Balla, Jozsef Balla, Christian Faul, Abolfazl Zarjou

**Affiliations:** 1 Division of Nephrology, Department of Medicine,; 2 Department of Genetics,; 3 Center for Neurodegeneration and Experimental Therapeutics, Department of Neurology, and; 4 Department of Pathology, University of Alabama at Birmingham, Birmingham, Alabama, USA.; 5 HUN-REN-UD Vascular Pathophysiology Research Group 11003, Division of Nephrology, Department of Internal Medicine, Faculty of Medicine, University of Debrecen, Debrecen, Hungary.

**Keywords:** Immunology, Nephrology, Cell stress, Chronic kidney disease, Macrophages

## Abstract

Macrophages, endowed with remarkable phenotypic plasticity, are essential for orchestrating injury responses and regulating iron homeostasis. Given the central role of ferritin heavy chain (FtH) as a molecular rheostat linking iron sequestration to redox-dependent signaling, we examined how myeloid FtH governs renal iron trafficking and ensuing oxidative stress pathways during acute kidney injury (AKI). Transcriptome analysis revealed coupling of FtH deficiency in monocytes and macrophages with activation of ferroptosis, a regulated cell death associated with iron accumulation. Moreover, myeloid FtH deletion worsened AKI, increasing leukocyte infiltration and iron deposition, together with ferroptosis-associated gene induction, oxidative stress, and lipid peroxidation. Notably, α-synuclein (SNCA), an iron-binding protein and the main pathological driver of Parkinson’s disease, was robustly induced both by FtH deficiency and following AKI. Mechanistic studies showed that monomeric SNCA exhibits ferrireductase activity, amplifying redox cycling and promoting ferroptotic cell death. Furthermore, SNCA expression was elevated in kidney pathologies characterized by leukocyte expansion in both mouse models and human cohorts, suggesting that inflammatory microenvironments promote SNCA accumulation and redox imbalance. These findings define a macrophage FtH/SNCA regulatory axis as a key driver of ferroptosis in AKI, implicating SNCA as a pathological nexus between iron dyshomeostasis and inflammatory kidney injury.

## Abstract 2

This study shows that a key iron-handling protein in immune cells controls kidney damage after injury by regulating iron, cell death, and a Parkinson’s-linked protein.

## Graphical abstract

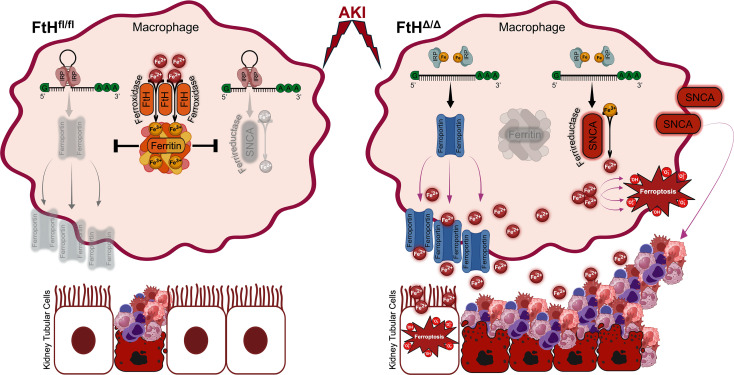

## Introduction

Acute kidney injury (AKI) is a prevalent clinical challenge contributing to considerable morbidity and affecting approximately one-quarter of hospitalized patients, with no currently available effective therapeutic interventions (1, 2). Following AKI, excessive tissue damage and maladaptive repair mechanisms are well-recognized drivers of chronic kidney disease (CKD) development and progression (3, 4). CKD is detrimental as it increases cardiovascular risk and reduces quality of life and life expectancy, leading to high mortality and morbidity rates while imposing a considerable financial burden on health care systems (5). Mechanistically, myeloid cells, particularly macrophages, play a pivotal role in orchestrating the response to tubular damage and facilitating repair (6, 7).

Despite the diverse etiologies of AKI, many cases exhibit an immune component involving macrophages (8, 9). Macrophages comprise a unique and heterogeneous population with remarkable phenotypic plasticity, enabling them to respond dynamically to a wide array of environmental signals. The extent of macrophage expansion observed in preclinical models and human biopsies directly correlates with the severity of kidney injury (9, 10). Moreover, macrophages are integral to maintaining iron homeostasis, a process increasingly recognized as central in kidney pathology (11, 12).

Iron is essential for aerobic life; however, when unrestrained, it catalyzes the formation of reactive oxygen species (ROS), leading to cellular and tissue injury and triggering a regulated form of cell death known as ferroptosis (13, 14). Consequently, balancing efficient iron distribution with mitigation of its potential toxicity is critical. Evolution has addressed this challenge with ferritin, a large intracellular nanocage capable of safely storing up to 4,500 iron atoms (15). Ferritin is a spherical protein complex composed of heavy and light chains (FtH and FtL). Through its ferroxidase activity, FtH converts the pro-oxidant ferrous iron (Fe^2+^) into the less reactive ferric form (Fe^3+^), thereby facilitating safe storage (15, 16). The indispensable role of FtH is underscored by the early embryonic lethality observed in mice with global FtH deletion, highlighting the paramount role of its ferroxidase activity (17).

Given their versatility and critical role in iron metabolism, macrophages are increasingly recognized as therapeutic targets across various disease settings to regulate iron availability, mitigate organ damage, and promote recovery (18). Building on this insight, we investigated the effects of myeloid-specific FtH deficiency on iron trafficking in kidney health and disease. We found upregulation of α-synuclein (SNCA) in macrophages in response to FtH deletion. SNCA is a small, intrinsically disordered protein primarily known for its role in neurodegenerative diseases, particularly Parkinson’s disease (19, 20). However, emerging evidence indicates that SNCA also contributes to immune regulation and possesses proinflammatory properties (21, 22). *Snca* is expressed in diverse cell types, with its highest expression in monocytes among peripheral leukocytes (23, 24). Additionally, SNCA’s expression and function are directly linked to iron metabolism. It is able to bind metals including iron, a process that facilitates its accumulation, and, in a self-propagating manner, accelerates tissue damage (25, 26). Moreover, monomeric SNCA possesses ferrireductase activity, potentially driving iron-mediated oxidative stress via generation of pro-oxidant ferrous iron (27, 28). In this context, SNCA appears to play a dual role, contributing to immune regulation by promoting a proinflammatory microenvironment while also influencing iron metabolism. This positions SNCA as a potentially key factor in inflammatory and degenerative diseases beyond the nervous system. However, the pathogenic role of macrophage SNCA expression in the context of kidney disease, and its interaction with FtH and iron, is not known and constitutes the focus of this report.

## Results

### Myeloid FtH deletion triggers SNCA induction in macrophages.

**Figure 1:**
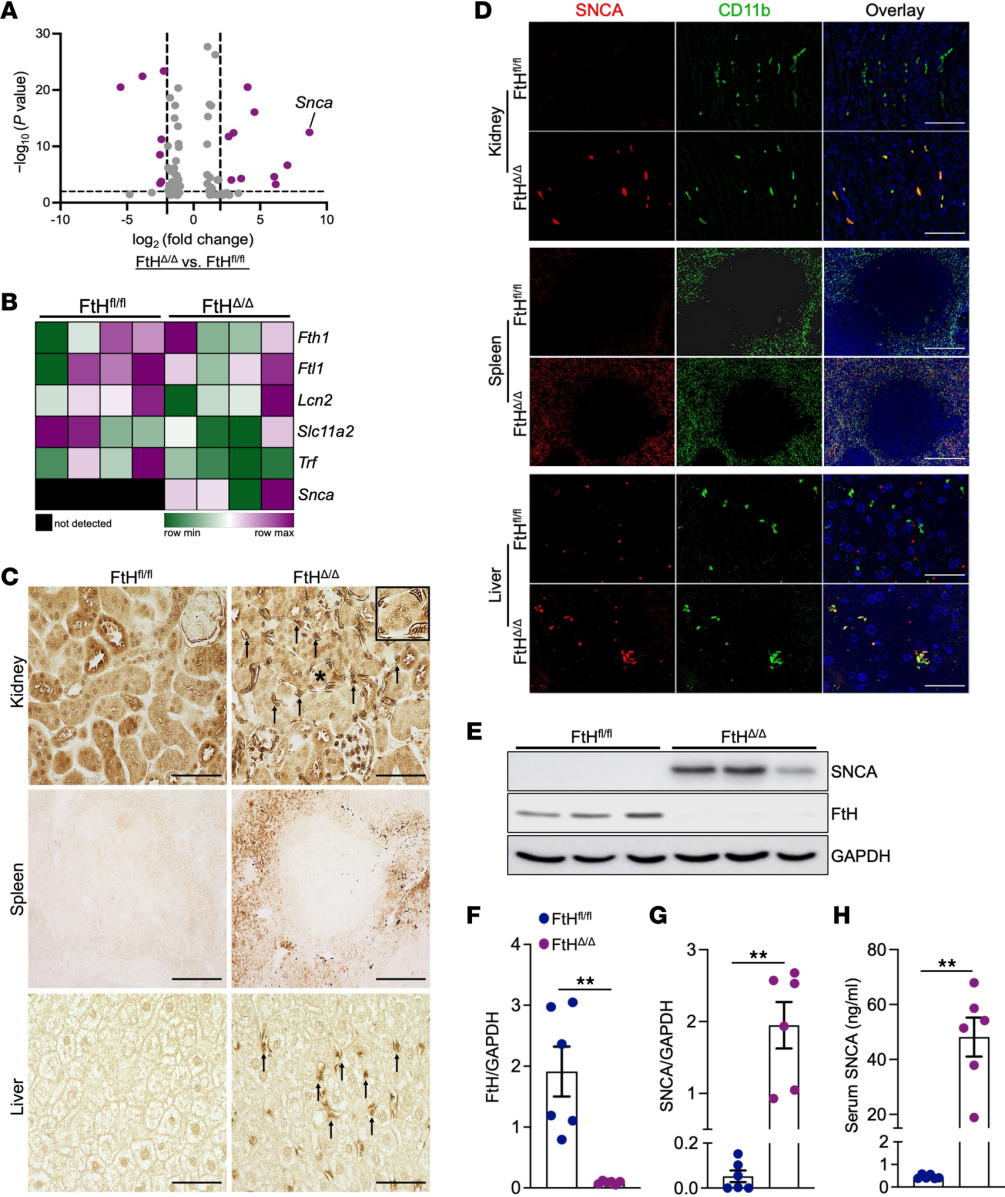
Myeloid FtH deletion triggers SNCA induction in macrophages. (**A**) Volcano plot showing differentially expressed genes in the kidneys of WT (FtH^fl/fl^) and myeloid-specific FtH-deficient (FtH^Δ/Δ^) mice under quiescent conditions following bulk RNA-seq (*n* = 4 per group). (**B**) Heatmap illustrating the expression of genes that encode iron-binding proteins in kidneys of FtH^fl/fl^ and FtH^Δ/Δ^ mice using normalized reads obtained via bulk RNA-seq. (**C**) Representative immunohistochemistry using an anti-SNCA antibody in the kidney, spleen, and liver of FtH^fl/fl^ and FtH^Δ/Δ^ mice under homeostatic conditions. Black arrows in the FtH^Δ/Δ^ kidney indicate SNCA-expressing interstitial cells. Inset: Higher magnification of a tubule with surrounding SNCA-expressing cells, marked with an asterisk. Scale bars: kidney, 100 μm; spleen, 200 μm; liver, 50 μm. (**D**) Immunofluorescence staining of SNCA and the myeloid marker CD11b in the kidney, spleen, and liver of FtH^fl/fl^ and FtH^Δ/Δ^ mice under baseline conditions. Scale bars: kidney, 25 μm; spleen, 200 μm; liver, 25 μm. (**E**) Representative Western blot of FtH and SNCA expression levels in FtH^fl/fl^ and FtH^Δ/Δ^ spleen at baseline. GAPDH was used as a loading control. (**F** and **G**) Densitometric analysis of spleen FtH and SNCA expression, normalized to GAPDH (*n* = 6 per genotype). (**H**) Serum SNCA levels measured by ELISA (*n* = 6 per genotype). **P < 0.01.

To explore the transcriptional alterations linked to FtH deletion in myeloid cells, we conducted an unbiased bulk RNA-seq analysis of the kidneys from WT mice (FtH^fl/fl^) and mice with myeloid-specific FtH deficiency (FtH^Δ/Δ^) under quiescent conditions. We identified multiple significantly modulated genes, with *SNCA* notably upregulated in the kidneys of FtH^Δ/Δ^ mice compared with controls (Figure 1A). Among genes that encode proteins with iron binding capacity, only *Snca* revealed significant upregulation in FtH^Δ/Δ^ kidneys when compared with FtH^fl/fl^ controls (Figure 1B and Supplemental Figure 1A; supplemental material available online with this article; https://doi.org/10.1172/jci.insight.196521DS1). We also assessed transferrin receptor 1 (*Tfrc*) expression and observed no significant differences between genotypes, while hepcidin levels were below the limit of detection (Supplemental Figure 1A). Further mRNA analysis of key iron regulatory genes in the livers and spleens of FtH^fl/fl^ and FtH^Δ/Δ^ mice was consistent with prior reports (29–33) that have characterized and utilized this transgenic line (Supplemental Figure 1, B and C). Similarly, serum iron levels did not differ significantly between genotypes under baseline conditions (Supplemental Figure 1D). Immunostaining for SNCA in the kidneys revealed a similar tubular staining pattern among genotypes, most prominent in the outer cortical stripe (Supplemental Figure 1E). In contrast, a higher number of SNCA-positive interstitial cells were observed in FtH^Δ/Δ^ kidneys compared with FtH^fl/fl^ controls, indicating elevated expression in tissue-resident macrophages (Figure 1C and Supplemental Figure 1E). This observation was further supported by the marked SNCA expression in the splenic red pulp and interstitial cells resembling hepatic Kupffer cells in FtH^Δ/Δ^ livers (Figure 1C).

To validate the monocytic lineage source of SNCA signal, we performed immunofluorescence double staining on kidneys, spleens, and livers of FtH^fl/fl^ and FtH^Δ/Δ^ mice by targeting SNCA and CD11b (Figure 1D). To confirm the source of SNCA upregulation following FtH deletion, we used 2 additional well-characterized monocyte/macrophage markers, Ly6C and F4/80. Consistent with the CD11b-SNCA costaining pattern, we observed marked SNCA upregulation in Ly6C^+^ cells within FtH^Δ/Δ^ kidneys, spleen, and liver (Supplemental Figure 2, A–D). Similarly, SNCA expression was confirmed in F4/80^+^ splenic red pulp macrophages of FtH^Δ/Δ^ mice (Supplemental Figure 2E), reinforcing that loss of FtH primes cells with monocytic lineage toward a heightened SNCA-expressing state. Neutrophils were ruled out as the source of SNCA upregulation using Ly6G immunostaining (Supplemental Figure 3). Furthermore, using a publicly available transcriptomic database, we confirmed dominant expression of *Snca* in monocytic cell lineage when compared with other leukocytes (Supplemental Figure 4). Using the spleen as a rich source of monocytes, we validated and quantified significant induction of monomeric SNCA in response to FtH deletion (Figure 1, E–G). Consistent with its characterization as a secretory protein (34, 35), we found that macrophage upregulation of SNCA was also associated with a substantial increase in serum levels in FtH^Δ/Δ^ mice (Figure 1H). These findings suggest that FtH deficiency drives aberrant SNCA overexpression and accumulation within macrophages in various tissues, including the kidneys.

### Myeloid FtH deficiency exacerbates AKI and accelerates AKI-to-CKD progression.

**Figure 2:**
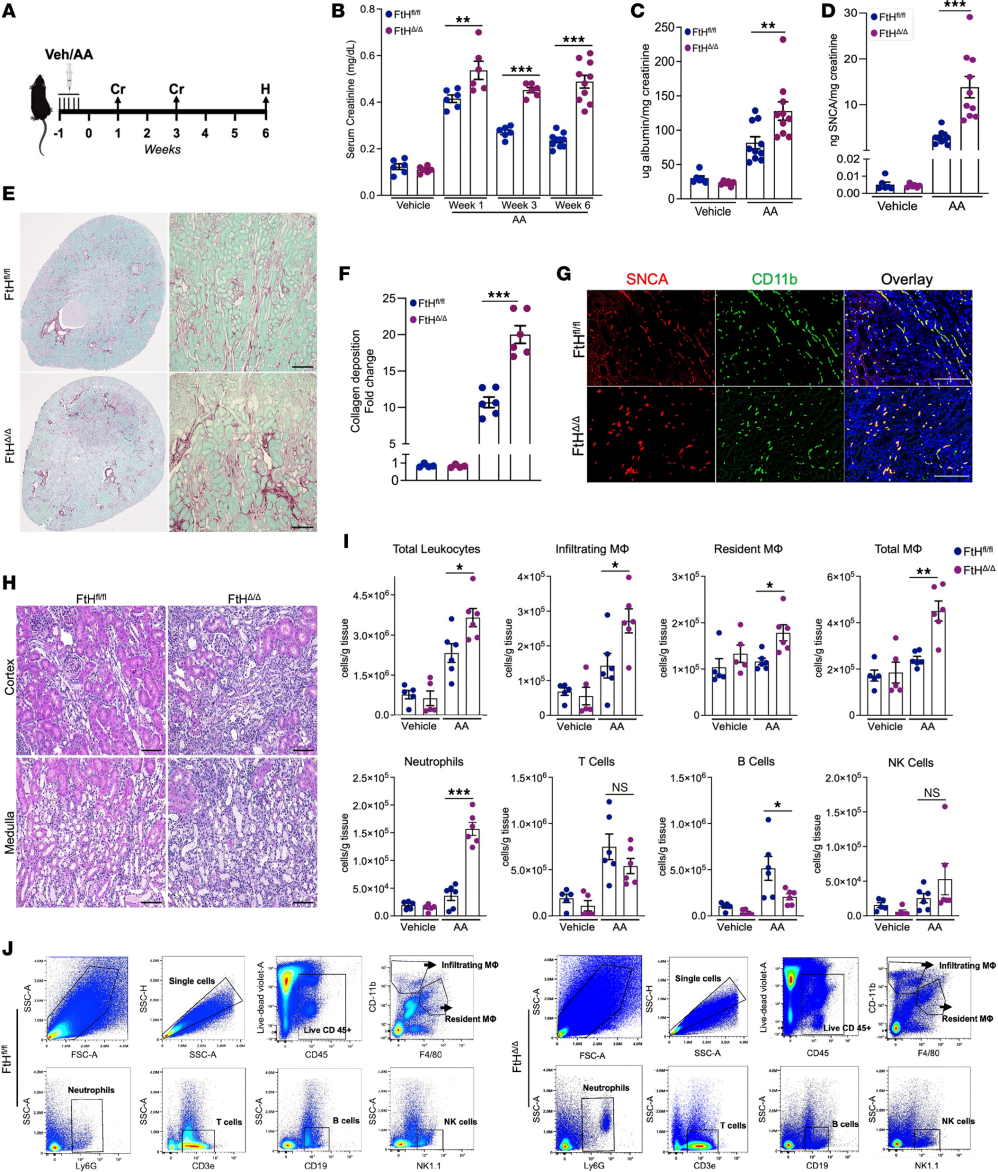
Myeloid FtH deficiency exacerbates AKI and accelerates AKI-to-CKD progression. (**A**) Schematic representation of the experimental design. Mice were given aristolochic acid (AA) or vehicle, with measurements and analyses performed at indicated time points. Cr, creatinine measurement; H, harvest and endpoint analyses. (**B**) Serum creatinine levels were measured at indicated time points in FtH^fl/fl^ and FtH^Δ/Δ^ mice (vehicle, *n* = 6 per genotype; AA, *n* = 6 per genotype for week 1 and week 3, *n* = 10 per genotype for week 6). (**C**) Albumin was measured in the urine of vehicle- and AA-treated mice and normalized to creatinine (vehicle, *n* = 6 per genotype; AA, *n* = 10 per genotype). (**D**) SNCA was measured in the urine of vehicle- and AA-treated animals and normalized to creatinine (vehicle, *n* = 6 per genotype; AA, *n* = 10 per genotype). (**E**) Representative sirius red/fast green stain demonstrating collagen deposition at 6 weeks after AA administration. Scale bars: 200 μm. (**F**) Quantification of kidney fibrosis (fold change) based on sirius red/fast green stain in FtH^fl/fl^ and FtH^Δ/Δ^ mice (vehicle, *n* = 4 per genotype; AA, *n* = 6 per genotype). (**G**) Representative immunofluorescence staining of kidney sections at 6 weeks after AA-induced injury analyzed for SNCA and CD11b, with DAPI nuclear counterstaining. Scale bars: 25 μm. (**H**) Representative H&E stain of FtH^fl/fl^ and FtH^Δ/Δ^ kidneys at week 6 after AA-induced CKD. Scale bars: 100 μm. (**I**) Quantification of immune cell populations in kidney tissues. Data are represented as number of cells per gram kidney tissue (vehicle, *n* = 5 per genotype; AA, *n* = 6 per genotype). (**J**) Representative gating strategy for identifying leukocytes (CD45^+^), macrophages (F4/80^+^), neutrophils (Ly6G^+^), T cells (CD3e^+^), B cells (CD19^+^), and NK cells (NK1.1^+^). NS, not significant; *P < 0.05, **P < 0.01, ***P < 0.001.

To elucidate the functional significance of myeloid FtH in kidney disease, we used a well-established and readily adjustable aristolochic acid–induced (AA-induced) model of AKI-to-CKD transition, characterized by pronounced tubular toxicity, robust inflammation, and substantial leukocyte accumulation. Mice from both genotypes received vehicle or AA, and kidney function was monitored via serial serum creatinine measurements (Figure 2A). Following the initial rise, WT mice showed a gradual decline in serum creatinine levels, although these values never fully returned to baseline (Figure 2B). In contrast, FtH^Δ/Δ^ mice exhibited significantly elevated serum creatinine levels at all time points compared with FtH^fl/fl^ controls, indicating that myeloid FtH deficiency leads to unresolved kidney injury (Figure 2B). Heightened CKD progression in FtH^Δ/Δ^ mice was further characterized by increased proteinuria, coupled with higher urinary SNCA excretion, and augmented collagen deposition (Figure 2, C–F). Next, we evaluated SNCA expression in kidney macrophages at 6 weeks after injury and found that nearly all CD11b^+^ cells from both genotypes expressed SNCA. This robust induction was also evident in WT animals, although the signal was markedly more pronounced in FtH^Δ/Δ^ kidneys (Figure 2G). Histological analysis of kidney sections at week 6 after AA injection revealed substantial leukocyte expansion in both genotypes, which was strikingly higher in FtH^Δ/Δ^ mice (Figure 2H). To determine the cellular basis of the intensified leukocyte expansion in FtH^Δ/Δ^ kidneys, we conducted flow cytometry analysis of kidney leukocyte populations. This analysis revealed that the augmented leukocyte counts in FtH^Δ/Δ^ kidneys following AA-induced injury were primarily driven by the expansion of macrophages and neutrophils (Figure 2, I and J). Taken together, these findings demonstrate that myeloid FtH deletion exacerbates kidney injury, impairs repair processes, promotes excessive leukocyte expansion, and increases SNCA deposition, culminating in accelerating kidney disease progression.

### Single-cell transcriptomic analysis of kidney leukocytes reveals ferroptosis induction following FtH deletion.

**Figure 3:**
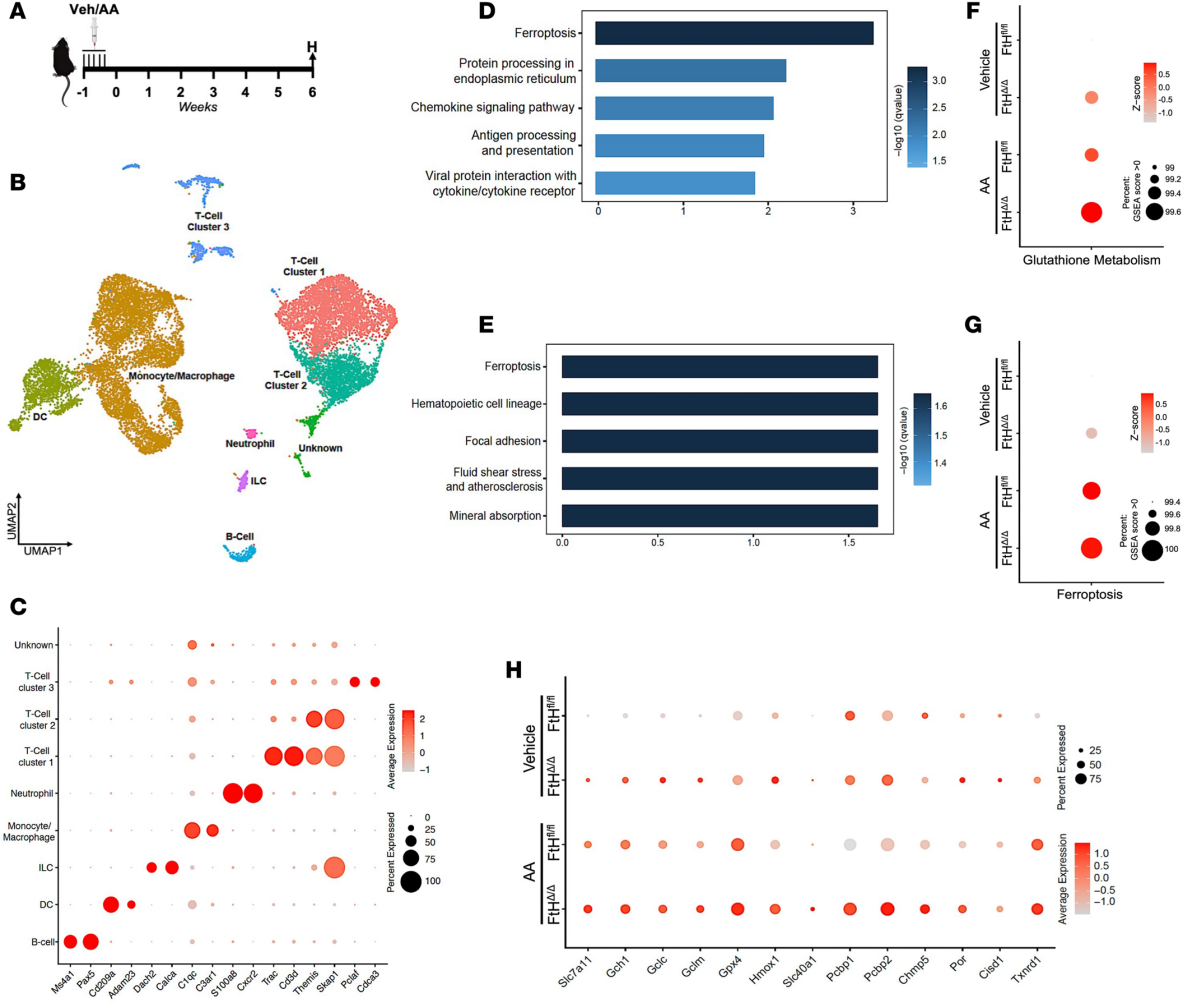
Single-cell transcriptomic analysis of leukocytes reveals ferroptosis induction following FtH deletion. (**A**) Schematic of the experimental setup. FtH^fl/fl^ and FtH^Δ/Δ^ mice received vehicle or AA for 5 consecutive days. Mice were harvested for scRNA-seq analysis of kidney leukocytes 6 weeks after vehicle or AA administration. H, harvest and endpoint analysis. (**B**) UMAP plot showing the clustering of immune cells (CD45^+^) in the kidney based on scRNA-seq. The analysis identifies 9 distinct clusters, with contaminating kidney cells and clusters representing less than 1% removed. (**C**) Cell type–specific expression of marker genes for manually annotated clusters. Dot size denotes percentage of cells expressing the marker. Color scale represents average gene expression values. (**D**) Pathway enrichment analysis of differentially expressed genes in FtH-deficient cells versus WT cells under vehicle-treated conditions. (**E**) Pathway enrichment analysis of differentially expressed genes in FtH-deficient cells versus WT cells following AA administration. (**F** and **G**) Gene set enrichment analysis (GSEA) for glutathione metabolism (**F**) and ferroptosis (**G**) pathways in monocytes/macrophages from vehicle- and AA-treated FtH^fl/fl^ and FtH^Δ/Δ^ mice. The size of the dots represents the percentage of genes enriched in the pathway, while the color indicates the *z* score. (**H**) Dot plot showing key genes involved in ferroptosis across different genotypes and experimental conditions. Dot size denotes percentage of cells expressing the marker. Color scale represents average gene expression values.

To examine the transcriptional response to FtH deletion, we performed scRNA-seq on kidney leukocytes isolated from FtH^fl/fl^ and FtH^Δ/Δ^ mice 6 weeks after AA or vehicle treatment (Figure 3A). CD45^+^ immune cells were sorted from kidneys and subjected to scRNA-seq using the 10x Genomics platform. Unbiased clustering of single-cell transcriptomic data identified 9 distinct immune cell populations (Figure 3B and Supplemental Figure 5). Analysis of the top differentially expressed genes confirmed the presence of all major immune cell types, including monocytes/macrophages, DCs, neutrophils, innate lymphoid cells (ILCs), B cells, and 3 distinct T cell clusters (Figure 3, B and C). Kyoto Encyclopedia of Genes and Genomes (KEGG) pathway analysis of FtH-deficient cells revealed ferroptosis as the most significantly activated pathway, both under quiescent conditions and following AA administration (Figure 3, D and E). Additionally, pathways associated with protein processing, chemokine signaling, and antigen presentation were enriched, highlighting a dysregulated immune response in the absence of FtH (Figure 3D). Given that Lyz2-Cre is expressed in immune cells beyond the monocytic lineage, we conducted a gene set enrichment analysis specifically on monocytes/macrophages to evaluate the enrichment of the glutathione metabolism pathway, rapidly induced during ferroptosis, as well as the ferroptosis pathway itself. This analysis confirmed the activation of both pathways in FtH-deficient monocytes/macrophages, regardless of injury state (Figure 3, F and G). This pattern was consistent when monocytes and macrophages were analyzed separately. Both cell populations displayed ferroptosis as the most prominently upregulated pathway in FtH^Δ/Δ^ mice following AA-induced injury. In addition, ferroptosis also ranked as the top KEGG-enriched pathway in FtH-deficient monocytes under vehicle-treated conditions, whereas homeostatic macrophages did not show significant enrichment for any specific pathway (Supplemental Figure 6, A–D). Moreover, the dot plot shows a robust induction of key ferroptosis-related genes in macrophages following FtH deletion, observed under both vehicle and AA treatment conditions (Figure 3H). These results infer that loss of FtH increases susceptibility of macrophages to iron metabolism perturbations and subsequent activation of ferroptosis in the kidney.

### Myeloid FtH deficiency confers susceptibility to ferroptosis following injury.

**Figure 4:**
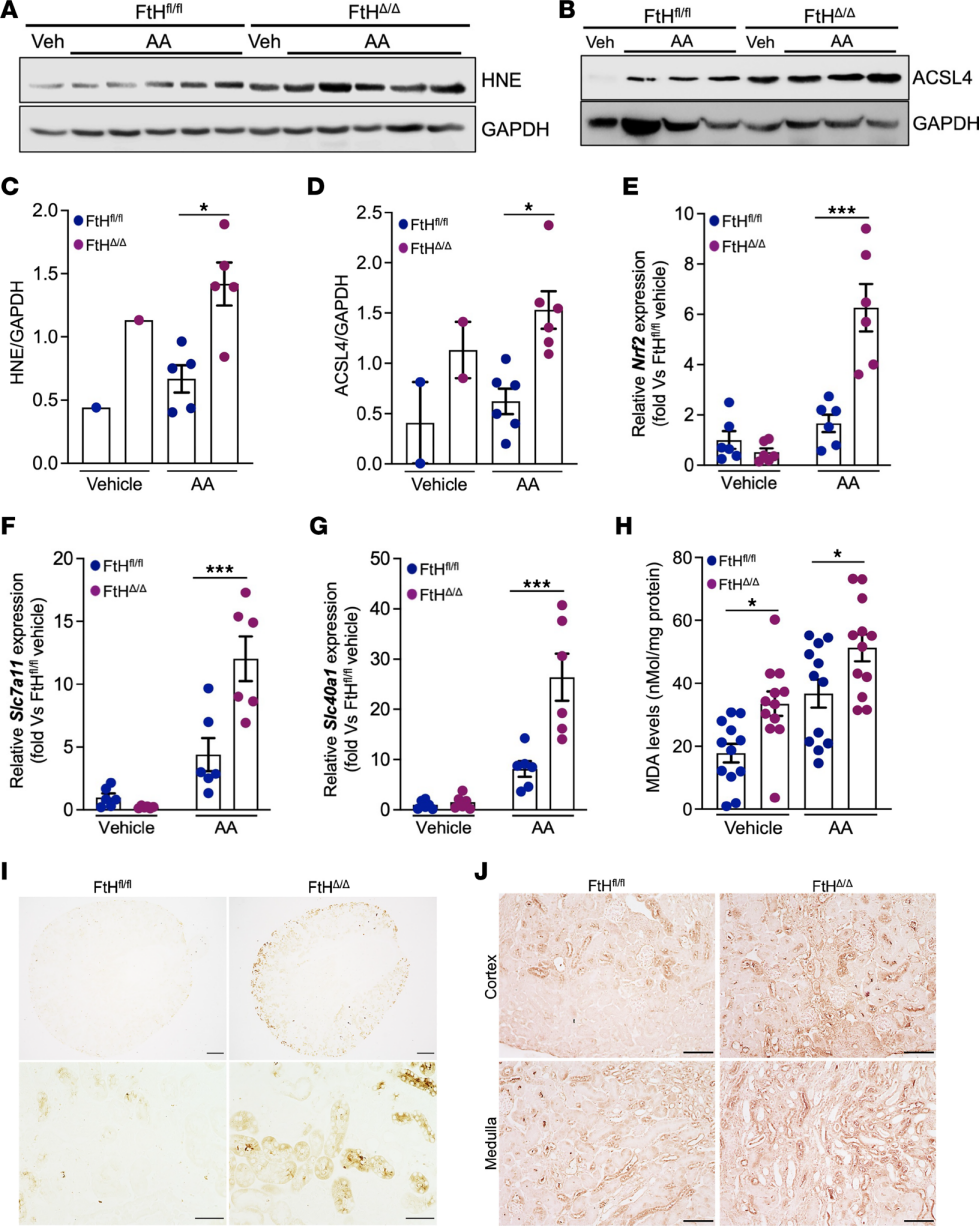
Myeloid FtH deficiency confers susceptibility to ferroptosis following injury. (**A** and **B**) Representative blot showing HNE (**A**) and ACSL4 (**B**) protein expression levels in kidney lysates from mice treated with vehicle or AA. (**C**) Densitometric quantification of HNE expression normalized to GAPDH (vehicle, *n* = 1 per genotype; AA, *n* = 5 per genotype). (**D**) Densitometric quantification of ACSL4 expression normalized to GAPDH (vehicle, *n* = 1 per genotype; AA, *n* = 6 per genotype). (**E**–**G**) mRNA expression levels of *Nrf2* (**E**), *Slc7a11* (**F**), and *Slc40a1* (**G**) in kidneys at 6 weeks after treatment. Data are normalized to *Gapdh* and represented as fold versus FtH^fl/fl^ vehicle group (vehicle, *n* = 6 per genotype; AA, *n* = 6 per genotype). (**H**) Whole-kidney lysates, collected at 6 weeks from both genotypes after vehicle or AA treatment, were analyzed to quantify malondialdehyde (MDA) levels as a measure of lipid peroxidation (*n* = 12 per genotype and treatment condition). (**I**) Modified Perls Prussian blue staining demonstrating iron deposition in kidneys of FtH^fl/fl^ and FtH^Δ/Δ^ mice at 6 weeks after AA treatment. Scale bars: 200 μm (top), 50 μm (bottom). (**J**) Representative immunohistochemistry to detect HNE accumulation in both genotypes at 6 weeks after AA administration. Scale bars: 100 μm. *P < 0.05, ***P < 0.001.

Given the wave-like propagation of ferroptosis within affected tissues, we reasoned that the exacerbated injury and CKD progression observed in FtH^Δ/Δ^ kidneys may be driven by increased ferroptotic activity. This deduction was examined by assessment of lipid peroxidation and ferroptosis-associated marker levels. Western blot analysis revealed significantly elevated levels of 4-hydroxynonenal (HNE), a by-product of lipid peroxidation, and acyl-CoA synthetase long-chain family member 4 (ACSL4), a key regulator of ferroptosis, in FtH^Δ/Δ^ kidneys compared with FtH^fl/fl^ controls (Figure 4, A–D). We also examined these patterns in kidneys under baseline conditions, and while a trend toward higher levels in FtH^Δ/Δ^ kidneys was observed, the difference did not reach statistical significance (Supplemental Figure 7). To further investigate the ferroptotic signaling cascade, we quantified the mRNA expression of other key regulatory genes. In FtH^Δ/Δ^ mice, nuclear factor erythroid 2–related factor 2 (*Nrf2*), a central antioxidant regulator rapidly activated during ferroptotic injury, was significantly upregulated after injury (Figure 4E). Similarly, *Slc7a11* and *Slc40a1* (ferroportin), which are critical for glutathione metabolism and iron export, respectively, were markedly upregulated (Figure 4, F and G). Subsequently, we quantified whole-kidney malondialdehyde levels after injury as a surrogate for lipid peroxidation, revealing a pronounced elevation in FtH^Δ/Δ^ mice relative to controls (Figure 4H). Correspondingly, we observed increased widespread accumulation of renal iron and HNE in FtH^Δ/Δ^ kidneys, hallmark features of ferroptosis activation (Figure 4, I and J, respectively). These findings support heightened ferroptosis in the kidneys of FtH^Δ/Δ^ mice and align with the well-documented wave-like propagation of ferroptosis once an initiating cell population is engaged.

### Excessive ferroptotic activity as the principal driver of kidney injury in FtH^Δ/Δ^ mice.

**Figure 5:**
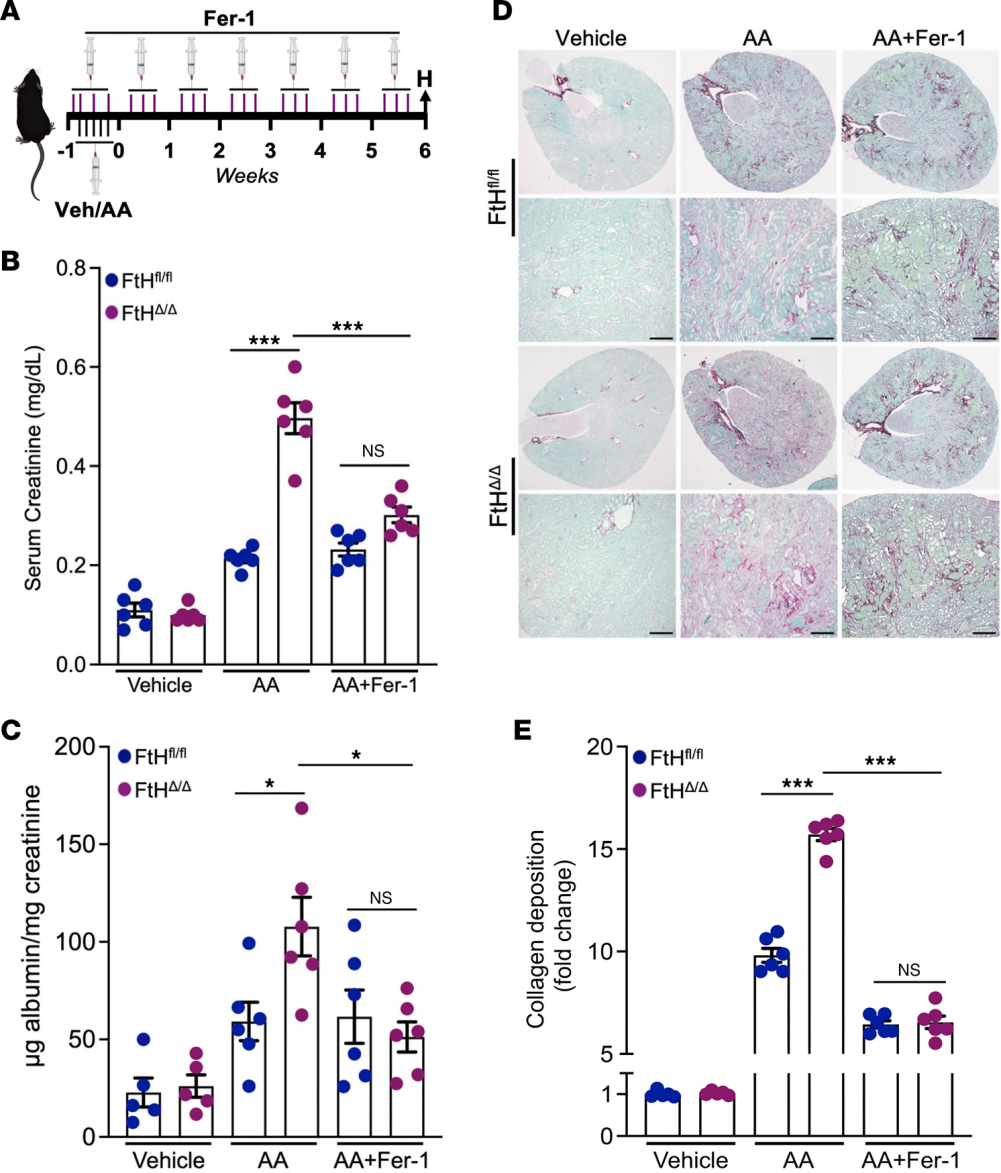
Excessive ferroptotic activity as the principal driver of kidney injury in FtH^Δ/Δ^ mice. (**A**) Schematic illustrating the experimental design for ferrostatin-1 (Fer-1) treatment to diminish ferroptosis-induced injury. (**B**) Serum creatinine levels across genotypes and experimental conditions at 6 weeks after indicated treatment (vehicle, *n* = 6 per genotype; AA, *n* = 6 per genotype). (**C**) Urinary albumin levels measured in vehicle-, AA-, and AA+Fer-1–treated mice and normalized to urine creatinine (vehicle, *n* = 6 per genotype; AA+Fer-1, *n* = 6 per genotype). (**D**) Representative sirius red/fast green staining demonstrating collagen deposition at 6 weeks after vehicle, AA, and AA+Fer-1 administration in FtH^fl/fl^ and FtH^Δ/Δ^ mice (vehicle, *n* = 6 per genotype; AA, *n* = 6 per genotype; AA+Fer-1, *n* = 6 per genotype). Scale bars: 200 μm. (**E**) Quantification of collagen deposition (fold change) based on fast green/sirius red stain in FtH^fl/fl^ and FtH^Δ/Δ^ mice after vehicle, AA, and AA+Fer-1 administration (vehicle, *n* = 6 per genotype; AA, *n* = 6 per genotype; AA+Fer-1, *n* = 6 per genotype). NS, not significant; *P < 0.05, ***P < 0.001.

To assess the functional impact of ferroptosis in this model, we administered ferrostatin-1 (Fer-1), a potent and specific ferroptosis inhibitor, at specific intervals before, during, and after AA-induced kidney injury (Figure 5A). Notably, Fer-1 treatment effectively reduced serum creatinine levels in FtH^Δ/Δ^ mice to levels comparable to those in FtH^fl/fl^ controls treated with AA alone or in combination with Fer-1 (Figure 5B). Concordantly, both the extent of albuminuria (Figure 5C) and the degree of collagen deposition (Figure 5, D and E) were indistinguishable between the two genotypes when treated with Fer-1. In contrast, AA exposure in the absence of ferroptosis inhibition elicited a significantly greater elevation in albuminuria (Figure 5C) and fibrosis (Figure 5, D and E) in FtH^Δ/Δ^ mice. Collectively, these findings indicate that ferroptosis plays a key role in the heightened kidney injury observed in FtH^Δ/Δ^ mice.

### Monomeric SNCA’s ferrireductase activity drives oxidative stress, triggering ferroptosis.

**Figure 6:**
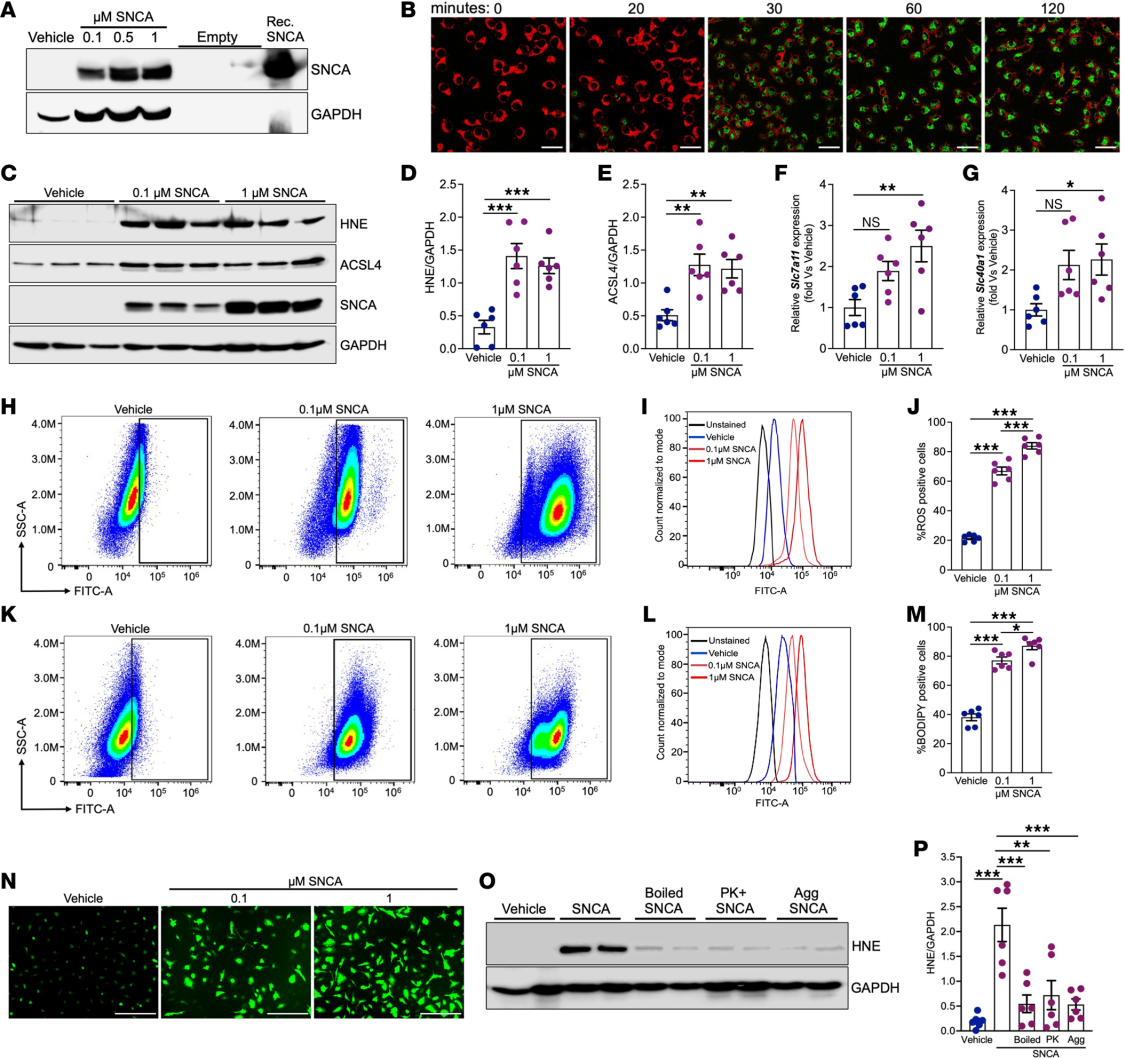
Monomeric SNCA’s ferrireductase activity drives oxidative stress, a hallmark of ferroptosis. (**A**) Representative Western blot showing dose-dependent uptake of recombinant monomeric SNCA by BMDMs after 16 hours of incubation. Recombinant SNCA was used as a positive control. (**B**) For uptake assays, BMDMs were treated with A488-labeled recombinant SNCA, and the A488 signal was monitored using live-cell imaging. The green signal indicates successful internalization of the recombinant monomeric SNCA. Scale bars: 100 μm. (**C**) Representative Western blot showing HNE and ACSL4 expression levels in BMDMs treated with vehicle or SNCA. (**D** and **E**) Densitometric quantification of HNE (**D**) and ACSL4 (**E**) expression (*n* = 6 for all treatment groups). (**F** and **G**) mRNA expression levels of *Slc7a11* (**F**) and *Slc40a1* (**G**) in BMDMs treated with vehicle or SNCA (*n* = 6 for all treatment groups). (**H**) Representative flow cytometry plot showing ROS detection using DCFDA in BMDMs treated with vehicle or monomeric SNCA. (**I**) Representative histogram of DCFDA fluorescence intensity. (**J**) Quantification of ROS-positive cells following SNCA treatment (*n* = 6 for all treatment groups). (**K**) Representative flow cytometry plot showing lipid peroxidation levels measured using BODIPY 581/591 in BMDMs treated with vehicle or monomeric SNCA. (**L**) Representative histogram of BODIPY fluorescence intensity. (**M**) Quantification of BODIPY-positive cells (*n* = 6 for all treatment groups). (**N**) Representative images demonstrating intracellular ROS using DCFDA in BMDMs treated with increasing concentrations of SNCA. Scale bars: 100 μm. (**O**) Representative Western blot demonstrates HNE expression in BMDMs treated with vehicle, monomeric SNCA, boiled monomeric SNCA, proteinase K–treated monomeric SNCA, or aggregated SNCA (fibrils) for 16 hours. (**P**) Densitometric quantification of HNE expression, normalized to GAPDH (*n* = 6 for all treatment groups). NS, not significant; *P < 0.05, **P < 0.01, ***P < 0.001.

To examine whether SNCA can directly contribute to oxidative stress and ferroptosis, we cultured bone marrow–derived macrophages (BMDMs) from WT mice and treated them with increasing concentrations of recombinant monomeric SNCA (0.1, 0.5, and 1 μM) for 16 hours. Western blot analysis confirmed a dose-dependent uptake of SNCA by BMDMs (Figure 6A). Live-cell imaging revealed rapid SNCA uptake that began within 20 minutes of incubation (Figure 6B).

To assess SNCA’s impact on lipid peroxidation, we used Western blot analysis to measure HNE and ACSL4 expression and demonstrate a significant increase in both markers following monomeric SNCA treatment (Figure 6, C–E). Additionally, quantitative real-time PCR analysis revealed upregulation of key ferroptosis-associated genes, *Slc7a11* and *Slc40a1*, upon SNCA exposure (Figure 6, F and G).

We subsequently examined the effect of SNCA treatment on generation of intracellular ROS. Using DCFDA staining and flow cytometry analysis, we observed a dose-dependent increase in ROS-positive cells following SNCA treatment compared with vehicle-treated cells (Figure 6, H–J). Lipid peroxidation, a characteristic by-product of ferroptosis, was assessed using the lipid peroxidation sensor BODIPY 581/591 and flow cytometry analysis. Notably, SNCA treatment led to a dose-dependent increase in lipid peroxidation, closely aligning with the observed rise in ROS levels (Figure 6, K–M). The dose-dependent increase in intracellular ROS burden following SNCA treatment was further visualized through fluorescent microscopy (Figure 6N). To validate the paramount role of SNCA’s ferrireductase activity in ROS generation, we used 3 approaches to diminish this activity: boiled SNCA, proteinase K–treated SNCA, and aggregated SNCA (fibrils), which loses its ferrireductase function as a result of improper protein folding. We found that only native monomeric SNCA, but not its aggregated or denatured forms, induced HNE accumulation (Figure 6, O and P). Next, we examined effects of monomeric SNCA on tubular ferroptotic susceptibility by using HK-2 cells, a human proximal tubular cell line. In contrast to BMDMs, SNCA alone did not substantially increase oxidative stress in HK-2 cells, as assessed by DCFDA fluorescence microscopy and HNE immunodetection (Supplemental Figure 8, A and B, respectively). However, co-exposure to SNCA with either iron or erastin produced a markedly amplified oxidative stress (Supplemental Figure 8, A and B). We reasoned that the heightened sensitivity of BMDMs to SNCA-induced ferroptotic signaling is attributable to their inherently robust phagocytic and endocytic capacity, which facilitates distinctly greater intracellular accumulation of exogenous SNCA. Consistent with this interpretation, under identical treatment conditions (1 μM SNCA), HK-2 cells internalized substantially lower amounts of this protein compared with BMDMs (Supplemental Figure 8C), supporting the conclusion that differential cellular uptake likely contributes to their divergent oxidative stress responses under conditions of SNCA excess. Together, these results indicate that monomeric SNCA promotes oxidative stress via its ferrireductase activity, contributing to lipid peroxidation and ferroptosis in BMDMs.

### SNCA accumulation is a hallmark of kidney diseases characterized by leukocyte expansion across species.

**Figure 7:**
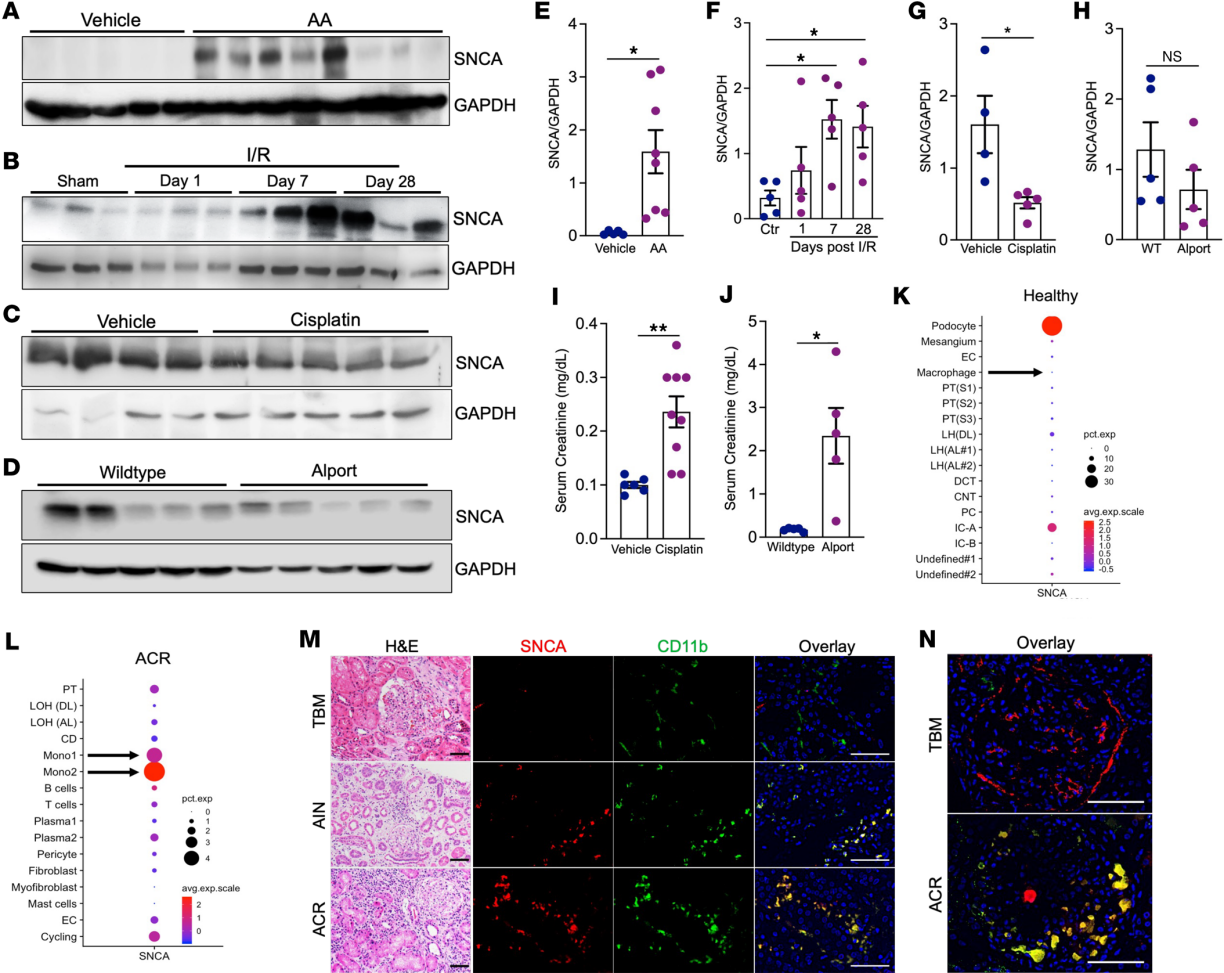
SNCA accumulation is a hallmark of kidney diseases marked by leukocyte expansion across species. (**A**–**D**) Western blot analysis of monomeric SNCA in kidney lysates from 4 different mouse models of kidney disease: (**A**) WT mice were treated with vehicle or AA. Kidney lysates were collected 6 weeks after AA administration. (**B**) WT mice were subjected to 20 minutes of ischemia/reperfusion (I/R) injury. Kidney samples were collected at indicated time points. (**C**) Protein expression of SNCA in WT kidneys treated with cisplatin as described in Methods. (**D**) SNCA expression in kidneys from WT (Col4a3^+/+^) and Alport (Col4a3^–/–^) mice at 12 weeks of age. (**E**–**H**) Densitometric analysis of SNCA protein expression in nephropathy models: AA (vehicle, *n* = 5; AA, *n* = 8) (**E**), I/R (sham, *n* = 5; days 1, 7, 28, *n* = 5 per time point) (**F**), cisplatin (vehicle, *n* = 4; cisplatin, *n* = 5) (**G**), and Alport syndrome (*n* = 5 per genotype) (**H**), normalized to GAPDH. (**I**) Serum creatinine levels in vehicle- and cisplatin-treated mice (vehicle, *n* = 6; cisplatin, *n* = 9). (**J**) Serum creatinine in WT and Alport mice measured at 12 weeks of age (*n* = 5 per genotype). (**K** and **L**) Analysis of *Snca* expression using publicly available scRNA-seq datasets from healthy (**K**) and rejected (ACR) (**L**) human kidney allografts. Arrows indicate monocyte/macrophage expression of *Snca* in healthy (**K**) and ACR (**L**) kidneys. (**M**) H&E stain (left) highlights histopathological features of human kidney biopsies from patients with TBM disease, acute interstitial nephritis (AIN), and acute cellular allograft rejection (ACR). Immunofluorescence staining of SNCA and CD11b validates overlap in AIN and ACR, but not in TBM. Scale bars: H&E stains, 100 μm; immunofluorescence, 25 μm. (**N**) Higher-magnification overlay images of a healthy-appearing glomerulus in TBM and a case of glomerulitis in ACR. Scale bars: 25 μm. NS, not significant; *P < 0.05, **P < 0.01.

To explore the dynamics of SNCA accumulation and its correlation with various kidney disease settings, we used 4 distinct models: ischemia/reperfusion (I/R) injury, AA- and cisplatin-induced nephropathy, and a mouse model of Alport syndrome (Col4a3^–/–^) with spontaneous progression of CKD. Western blot analysis revealed a significant increase in SNCA protein levels in the kidneys of mice subjected to AA-induced nephropathy at 6 weeks compared with vehicle-treated controls (Figure 7A). Similarly, in the I/R injury model, SNCA levels progressively increased over time, with the most pronounced upregulation observed at days 7 and 28 after injury (Figure 7B). In contrast, kidneys from cisplatin-treated mice and mice that mimic Alport syndrome did not show accumulation of SNCA (Figure 7, C and D). Densitometric quantification confirmed a significant upregulation of SNCA in both the AA and I/R models (Figure 7, E and F). Conversely, SNCA expression was reduced in the cisplatin model, which is characterized by tubular toxicity and leukopenia, while no change was observed in the Alport mouse model (Figure 7, G and H). As expected, Western blot analysis of whole-kidney lysates demonstrated upregulation of FtH across all 4 models, consistent with its adaptive cytoprotective response during injury (Supplemental Figure 9, A–D). FtH kidney injury of all 4 models was confirmed by serum creatinine measurements (Figure 2B and Figure 7, I and J).

To investigate the translational and clinical implications of macrophage SNCA expression in human kidney disease, we analyzed publicly available single-nucleus RNA-seq datasets from healthy human kidneys and kidney allografts with acute cellular rejection (ACR). In healthy kidneys, SNCA expression was predominantly restricted to podocytes, with minimal expression detected in macrophages (Figure 7K). However, in ACR kidneys, marked by heavy leukocyte expansion, SNCA expression was markedly upregulated in monocytes, emerging as their primary source of expression (Figure 7L). These results align with our supplemental data, which demonstrate SNCA enrichment in monocytic-lineage cells during human AKI (Supplemental Figure 10 and Supplemental Tables 1 and 2). In agreement with these findings, analysis of human diabetic kidney disease, which is largely devoid of substantial leukocyte expansion, showed no significant SNCA enrichment in leukocytes (Supplemental Figure 11). These findings prompted us to further investigate the cellular localization of SNCA in human kidney diseases with varying degrees of leukocyte involvement, including thin basement membrane disease (TBM), where pathogenesis is largely independent of leukocyte expansion, and acute interstitial nephritis (AIN) and ACR, which are heavily leukocyte dependent (Supplemental Table 3). Routine histochemical immunostaining, along with electron microscopy, was used by the pathologist to confirm the primary diagnosis (Figure 7M and Supplemental Figure 12). Immunofluorescence double staining revealed a lack of SNCA expression in CD11b^+^ cells in the TBM setting, whereas a pronounced upregulation of SNCA colocalized with CD11b^+^ cells in diagnoses of AIN and ACR (Figure 7M). This finding was further reinforced by the presence of severe tubulitis and glomerulitis in ACR (Figure 7, M and N). These results suggest that kidney diseases marked by leukocyte, particularly macrophages, expansion create a unique milieu that fosters SNCA accumulation. This underscores the potential for kidney diseases marked by leukocyte expansion to serve as a systemic driver of neurodegenerative processes, bridging inflammatory and iron-related pathologies across organ systems.

## Discussion

In this study, we demonstrate that deletion of FtH in macrophages is associated with the upregulation of SNCA, an iron-binding protein with ferrireductase activity that is implicated in the pathogenesis of several neurodegenerative disorders, including Parkinson’s disease (19, 20, 26). Following induction of AKI, FtH-deficient mice exhibited accelerated kidney disease progression accompanied by increased leukocyte expansion. Further analyses confirmed that activation of ferroptosis contributed to the injury observed in FtH^Δ/Δ^ mice. Mechanistically, our findings suggest that the ferrireductase activity of monomeric SNCA promotes oxidative stress, thereby increasing lipid peroxidation and promoting ferroptosis. Importantly, monomeric SNCA induction in macrophages emerges as a defining feature of kidney diseases marked by pronounced leukocyte aggregation, a pattern conserved across both murine models and human datasets.

AKI and its progression to CKD are a multifactorial disorder that, beyond its adverse impact on physiological benchmarks, triggers maladaptive crosstalk between the kidney and other organs, exacerbating inflammation, oxidative stress, and cardiovascular pathology, ultimately driving high morbidity and mortality (5, 36, 37). Dysregulated iron metabolism is a common feature in AKI and is frequently observed during the clinical progression of CKD (12, 38, 39). Additionally, aberrant kidney iron deposition and ferroptosis have been identified as key drivers of AKI pathogenesis and the transition from AKI to CKD (13, 40, 41). First described in 2012 (42), ferroptosis is a distinct and inherently iron-dependent form of regulated cell death characterized by extensive iron accumulation and lipid peroxidation during the cell death process (43). Various ferroptosis-inducing factors can overwhelm the glutathione metabolism pathway, leading to an exhaustion of antioxidant capacity and ultimately an oxidative imbalance resulting in cellular damage and death (43). Interestingly, ferroptosis can propagate across cell populations in a synchronized wave-like fashion, leading to a characteristic spatiotemporal pattern of cell death, referred to as a “wave of death” (44–47). This process is likely intensified by increased *Slc40a1* expression, which promotes the efflux of ferrous iron from cells undergoing ferroptosis, coupled with the release of iron-containing heme moieties from dying cells (48). Guided by our findings, we posit that FtH-deficient macrophages constitute the principal ferroptosis-engaging population in this model and likely serve as the initiating cellular source that propagates the earliest wave of ferroptotic signaling. This premise is supported by evidence demonstrating that ferroptosis is the most significantly enriched cellular pathway in FtH-deficient cells. This enrichment is accompanied by upregulation of markers associated with ferroptosis-mediated damage and activation of key ferroptosis regulators in whole kidneys. Importantly, experiments using Fer-1, a ferroptosis inhibitor (49), confirmed that the excess injury observed in FtH^Δ/Δ^ mice is primarily driven by ferroptosis. These findings further indicate that the pathological role of macrophage FtH in kidney injury is primarily dependent on the mode of injury, with leukocyte-rich, inflammation-driven models providing a more suitable context for defining its function than injury paradigms dominated by tubular cytotoxicity.

At the cellular level, regulation of key iron homeostasis proteins is orchestrated by iron regulatory proteins (IRPs) through their physical interaction with iron-responsive elements (IREs) (50). These cytoplasmic IRPs bind with high affinity to conserved hairpin structures located in the untranslated regions of mRNAs (IREs), allowing cells to delicately balance the expression of iron import, storage, and export proteins in response to fluctuations in intracellular iron levels (50). Notably, the expression of *Snca*, similarly to that of *FtH*, is controlled post-transcriptionally, as both mRNAs contain structured IREs in their 5′-untranslated regions that govern translation (51, 52). Specifically, the *Snca* IRE is formed at the splice junction of the first 2 exons of the *Snca* gene (53). Based on existing evidence (54, 55), and our findings, we propose that in the absence of FtH, an increase in the intracellular labile iron pool triggers the dissociation of IRPs from *Snca* mRNA, thereby enhancing its translation. However, contrary to ferroxidase activity of FtH that converts the unstable ferrous form of iron into a more stable ferric form (16), SNCA via its ferrireductase activity may augment oxidative stress by generating excess iron in ferrous state (27). While the exact function of neuronal SNCA continues to be debated, its abundant presence and ferrireductase activity in red blood cells are proposed to play a crucial role in maintaining iron in its ferrous state within hemoglobin, which is essential for efficient oxygen binding (56). Similarly, within macrophages, SNCA can contribute to generation of ROS, a fundamental process required for elimination of invading pathogens and coordination of signal transduction and cellular processes (57). Moreover, given that Slc40a1 exclusively exports iron in its ferrous state, it can be reasonably projected that SNCA’s ferrireductase activity is essential for facilitating macrophage iron export. An expanding body of evidence increasingly substantiates the induction of SNCA during inflammatory processes, highlighting its involvement in immune regulation (24). Moreover, SNCA has been identified as a potent chemoattractant, a critical modulator of dendritic cell phenotypic maturation, and a pivotal immunomodulator, thus playing a central role in the orchestration of innate immunity and the maintenance of overall immune competence (21, 24, 58, 59). Consistent with these findings, we observed an enrichment of inflammatory pathways in FtH-deficient monocytes under quiescent conditions, along with an increased leukocyte presence in the kidneys of FtH^Δ/Δ^ mice, which may, at least in part, be attributed to elevated SNCA levels.

Because of its molecular weight of 14 kDa, SNCA can traverse the glomerular filtration barrier and be excreted in the urine. Indeed, it was recently revealed that kidneys rapidly remove circulating SNCA, a process that declines with worsening kidney disease, leading to SNCA accumulation and ensuing aggregation into fibrils within the kidneys (60). These fibrils subsequently propagate to the brain, a process that is blocked by kidney denervation (60). While population studies indicate an elevated risk of Parkinson’s disease in patients with advanced CKD (61–64), the mechanisms that underpin this association remain unclear, particularly as most individuals with CKD do not develop Parkinson’s disease. This report offers a potential explanation for this discrepancy. We validate upregulation of SNCA in macrophages across species in response to kidney injury. In mice, we demonstrate kidney accumulation of monomeric SNCA in injury models characterized by predominant leukocyte expansion, such as I/R and AA. Conversely, kidney diseases with minimal leukocyte involvement in their pathogenesis, such as cisplatin-induced nephropathy and COL4A3 deficiency (Alport syndrome), do not exhibit increased SNCA levels in kidneys. Similarly, while we observed increased presence of macrophage SNCA in AIN and ACR, two kidney disease entities marked by a high degree of leukocyte aggregation, such expression was absent in TBM disease. Moreover, through analysis of publicly available transcriptome datasets, we confirm that *Snca* expression in macrophages is minimal under both healthy conditions and diabetic injury but is notably elevated in ACR. Collectively, our findings suggest that the etiology of CKD, along with the extent of leukocyte (particularly macrophage) accumulation, may predispose the kidneys to SNCA aggregation. This, in turn, could render this subgroup of patients more susceptible to subsequent brain accumulation of SNCA via propagation from the kidneys.

In summary, our study uncovers a mechanistic pathway linking FtH deficiency in macrophages to heightened kidney injury via a macrophage SNCA/FtH regulatory axis that governs ferroptosis signaling. Myeloid FtH deletion and consequent perturbation of iron homeostasis foster excessive iron accumulation, which in turn precipitates enhanced ferroptotic cell death in the kidney. Importantly, the elevated expression of SNCA in macrophages correlates with kidney disease models characterized by marked leukocyte aggregation and may explain, at least in part, the observed association between CKD and an increased risk of neurodegenerative disorders. These findings position the macrophage FtH/SNCA regulatory axis as a mechanistic fulcrum whereby perturbations in iron homeostasis drive inflammatory kidney pathology and may serve as a peripheral conduit for excess SNCA dissemination to the central nervous system, potentially contributing to synucleinopathy development.

## Methods

### Sex as a biological variable.

Only male mice were used in this study because of the well-established relative resistance of female mice to kidney injury and CKD progression.

### Antibodies and oligonucleotide sequences.

All primary antibodies and corresponding dilutions and primer sequences used in this study are summarized in Supplemental Tables 4 and 5.

### Mice.

FtH-floxed mice (FtH^fl/fl^) mice (65) were crossed with lysozyme 2–Cre mice, generating littermates that were either FtH^fl/fl^ or FtH^Δ/Δ^ on a C57BL/6 background. WT C57BL/6 mice were purchased from The Jackson Laboratory, and the colony was maintained at the University of Alabama at Birmingham (UAB). All mice were maintained in temperature-controlled rooms on a 12 hour light/12 hour dark cycle and were provided with food and water ad libitum. The age of all experimental mice was between 8 and 12 weeks.

### Aristolochic acid model of kidney injury.

FtH^fl/fl^ and FtH^Δ/Δ^ mice were randomly assigned to either the aristolochic acid (AA) group or the control group. Mice in the AA group received intraperitoneal injections of AA (2 mg/kg body weight; Sigma-Aldrich, A9451) once daily for 5 consecutive days to induce kidney injury, while control mice received an equal volume of saline as a vehicle.

### Ischemia/reperfusion model of AKI-to-CKD progression.

WT mice underwent a bilateral ischemia/reperfusion (I/R) renal injury procedure (20 minutes ischemia time) to induce AKI-CKD transition as previously described (66). We used samples from a previous study in which temporal serum creatinine measurements were published (66).

### Cisplatin model of CKD.

The cisplatin model of CKD was established as previously described (66). Briefly, 9 mg/kg body weight cisplatin (1.0 mg/mL solution in sterile 0.9% saline) was administered intraperitoneally once a week for 4 weeks to induce moderate CKD, and tissues were harvested 1 week after final injection. Control mice received saline as vehicle.

### Model of Alport syndrome.

Col4a3^+/+^ (WT) and Col4a3^–/–^ (Alport) mice were harvested at 12 weeks of age, at which point substantial evidence of advanced CKD is present (67).

### Ferrostatin-1 administration to mitigate ferroptosis.

FtH^fl/fl^ and FtH^Δ/Δ^ mice were intraperitoneally injected with 1 mg/kg ferrostatin-1 (Fer-1; Selleck Chemicals, S7243) on day –1, one day before the first AA injection. After the first AA administration, mice continued to receive Fer-1 injections 3 times a week.

### Kidney function measurement.

Serum and urine creatinine was quantified at the UAB-UCSD O’Brien Center for AKI Research using liquid chromatography–tandem mass spectrometry as previously described (68).

### Western blot analysis.

Tissues and cells were homogenized, and protein was isolated as previously described (66). Protein concentrations were measured by bicinchoninic acid (BCA) assay kit (Thermo Fisher Scientific, PI23227), and 25 to 50 μg protein samples were denatured at 95°C for 5 minutes for standard Western blots, or at 50°C for 30 minutes for SNCA Western blots, and then loaded on a 12% electrophoresis gel. Western gels were then transferred onto 0.45 μm PVDF transfer membranes (Thermo Fisher Scientific, 88518). After transfer, SNCA Western blots were incubated for 30 minutes in 0.4% paraformaldehyde in 1× PBS before blocking for 1 hour in 6% nonfat dry milk in PBS with 0.1% Tween 20 (PBST). All other blots were immediately blocked in 6% nonfat dry milk in PBST for 1 hour after transfer. After blocking, membranes were then incubated overnight at 4°C in 1% nonfat dry milk in PBST with primary antibody. Anti-rabbit or anti-mouse HRP-conjugated secondary antibodies (Jackson ImmunoResearch Laboratories, 115-035-003 and 111-035-003) were applied at 1:2,500 dilutions in 5% nonfat dry milk in PBST for 1 hour at room temperature. HRP-conjugated antibodies were detected via chemiluminescence using Ultra Digital-ECL Substrate Solution (Kindle Biosciences, R1002) and developed with the KwikQuant Pro Imager (Kindle Biosciences, D1010). Specificity of monomeric SNCA bands was confirmed in preliminary studies using brain tissue and recombinant SNCA as positive controls. Densitometry analysis was performed using ImageJ 1.54k (NIH), and results were normalized to GAPDH. Additional Western blots used for densitometry represent independent samples and are provided, along with representative blots, as a supplemental file.

### Quantitative real-time PCR.

Total RNA was extracted from cells and tissues using TRIzol (Invitrogen, 15596018). cDNA was synthesized from total RNA with the QuantiTect reverse transcription kit (QIAGEN, 205313) per the manufacturer’s instructions. Quantitative PCR was performed using PowerUp SYBR Green Master Mix (Applied Biosystems, A25777) on an Applied Biosystems StepOnePlus Real-Time PCR System. Quantification of gene expression was done via the 2^–ΔΔCt^ method, and *Gapdh* expression was used for normalization. All real-time primers can be found in Supplemental Table 5. All reactions were performed in triplicate, and specificity was monitored using melting curve analysis.

### Immunohistochemistry staining.

Immunohistochemistry staining was performed as previously described (69). Kidneys were fixed in 10% neutral-buffered formalin for 24 hours and then embedded in paraffin. Five-micrometer kidney sections were deparaffinized in xylenes, rehydrated in a series of ethanol rinses from 100% to 70% ethanol, then washed in distilled water. Antigen retrieval was performed in Trilogy (Cell Marque, 922P-06) at 95°C for 30 minutes. Sections were washed in distilled water and incubated in 3% H_2_O_2_ for 20 minutes followed by blocking in a buffer containing 1% BSA (Thermo Fisher Scientific, J64100-A1), 0.2% nonfat dry milk, and 0.3% Triton X-100 (Thermo Fisher Scientific, A16046-AE) in 1× PBS for 30 minutes. The primary antibody, SNCA (Cell Signaling, 4179S), was diluted 1:200 in the blocking buffer and incubated overnight at 4°C. Sections were washed once with PBST and twice with PBS for 5 minutes each. Anti-rabbit HRP-conjugated secondary antibody (Jackson ImmunoResearch Laboratories, 115-035-003) was diluted 1:500 and applied for 1 hour at room temperature. Sections were washed once with PBST and twice with PBS for 5 minutes each. Chromogen substrates (Vector Laboratories, SK-4103) were mixed per the manufacturer’s instructions and added to sections. Images were captured on a BZ-X700 All-In-One Fluorescence Microscope (Keyence).

### Immunofluorescence staining.

Deparaffinization, rehydration, antigen retrieval, and blocking were similar to the immunohistochemistry protocol outlined above. Sections were then incubated overnight at 4°C with primary antibodies (dilutions in Supplemental Table 4) diluted in blocking buffer. Sections were washed once with PBST and twice with PBS for 5 minutes each and then incubated with fluorescently tagged secondary antibodies (Supplemental Table 4) at 1:500 dilutions in blocking buffer. Sections were washed once with PBST and twice with PBS for 5 minutes each, followed by application of autofluorescence eliminator reagent (MilliporeSigma, 2160) per the manufacturer’s instructions. Slides were then mounted with DAPI (Invitrogen, P36962). Images were captured on a BZ-X700 All-In-One Fluorescence Microscope (Keyence). Notably, the very intense staining of SNCA in macrophages required us to decrease the intensity of staining to better depict these macrophages at the expense of tubules. Brightness and contrast adjustments were made in an identical manner for matched sections and were applied to the entire image.

### Sirius red/fast green stain.

Sirius red/fast green stain was performed on the tissue sections using a sirius red/fast green collagen staining kit (Chondrex, 50-152-6960) per the manufacturer’s instructions. Four images per kidney were acquired at a ×10 magnification with a BZ-X700 All-in-One Fluorescence Microscope. Percentage area positive and intensity (integrated density) of sirius red stain, as a measure of collagen deposition and fibrosis, were measured with ImageJ using the Triangle auto-threshold method. Color, brightness, and contrast adjustments were made in an identical manner for matched sections.

### Iron staining.

To enhance iron deposition visualization, the modified Perls stain method was implemented. Briefly, paraffin-embedded 5 μm kidney sections were deparaffinized in xylenes and rehydrated as mentioned above. Sections were blocked in 1% H_2_O_2_ for 10 minutes and then washed with distilled water. Sections were then placed in a working solution of distilled water containing 4% HCl and 4% potassium ferrocyanide for 60 minutes at room temperature and rinsed in tap water, and chromogen substrate (Cell Signaling, 8059S) was applied.

### Serum iron measurement.

Serum iron was measured with a Quantichrome iron assay kit (BioAssay systems) following the manufacturer’s instructions.

### Flow cytometry and fluorescence-activated cell sorting.

Leukocytes (CD45^+^ cells) were isolated from kidneys as previously described (70). Mice were anesthetized with isoflurane and perfused through the left ventricle with 10 mL of cold PBS. Kidneys were then removed, stripped of the capsule, weighed, and minced with a razor blade on a glass slide. The minced tissue was placed in 2 mL of DMEM containing 1.67 Wünsch units/mL Liberase (Roche, 5401119001) and incubated at 37°C for 30 minutes. Digestion was halted by addition of cold PBS containing 1% BSA. The resulting suspensions were passed through an 18-gauge needle 3 times and subsequently filtered through a 40 μm strainer. The samples were then centrifuged at 400*g* for 7 minutes, and the supernatant was aspirated. Red blood cells were lysed using ACK lysis buffer for 2 minutes, followed by washing with ice-cold PBS to obtain the remaining leukocytes. Cells were then stained with a violet fixable viability dye (Invitrogen, L34964) for 20 minutes in the dark at room temperature and treated with an unlabeled anti-CD16/32 antibody (BioLegend, 156604) to block FcγIII receptors for 30 minutes. Subsequently, cells were stained with the following antibodies: anti-CD45.2–Brilliant Violet 650 (BioLegend, 09836), anti-CD11b–Super Bright 600 (M1/70, Invitrogen), anti-F4/80–APC–eFluor 780 (Invitrogen, 63011282), anti-NK1.1–PE–C7 (Invitrogen, 12-5941-83), anti-Ly6G–Alexa Fluor 700 (BioLegend, 127622), anti-CD19–Super Bright 702 (BioLegend, 67-0193-82), and anti-CD3e–PE–Cy7 (Invitrogen, 25-0031-82). After staining, samples were washed and resuspended in 500 μL of staining buffer before analysis by flow cytometry using the Cytek Northern Lights instrument and SpectroFlo software. Single-color controls were used for spectral unmixing, and fluorescence-minus-one (FMO) controls were applied to set positive gates. Data were analyzed using FlowJo software (BD Biosciences/Waters Biosciences).

### Bulk RNA-seq data processing and analysis.

Total RNA was isolated from kidneys using TRIzol. RNA quality was assessed using the Agilent 2100 Bioanalyzer, and only samples with an RNA integrity number greater than 8.0 were used for RNA-seq. RNA was sequenced on a NextSeq 500 system (Illumina), and the library was prepared with the Agilent SureSelect Stranded mRNA kit. STAR (version 2.7.7a) was used to align the raw RNA-Seq FASTQ reads to the mouse reference genome (GRCm38 p6, Release M25) from Gencode (71). After alignment, HTSeq-count (version 0.11.3) was used to count the number of reads mapping to each gene (72). Normalization and differential expression were then applied to the count files using DESeq2 (73). Heatmaps were generated using the Morpheus platform (https://software.broadinstitute.org/morpheus/).

### scRNA-seq.

CD45^+^ immune cells were isolated by fluorescence-activated cell sorting (FACS) from the kidneys of vehicle- and AA-treated mice at 6 weeks after final injection, following the flow cytometry protocol described earlier. CD45^+^ cells were sorted on a BD FACSAria II at the UAB Flow Cytometry and Single Cell Core Facility. Isolated cells were then processed using the Chromium 3′ Single Cell RNA sequencing kit (10x Genomics) in accordance with the manufacturer’s protocol. Cell-specific barcoded sequencing libraries were generated, and sequencing was performed on an Illumina NovaSeq6000. Reads were processed using the 10x Genomics Cell Ranger Single-Cell Software Suite (v6.0). Each sample of scRNA-seq was mapped to the mouse reference genome (GRCm38) provided by 10x Genomics. The samples then were merged by loading of the Cell Ranger output h5 file using Seurat (v5.1) for data preprocessing, integration, and normalization. Cells with fewer than 200 genes, more than 6,000 unique molecular identifiers (UMIs), or more than 10% mitochondrial UMIs were excluded. RNA expression was normalized by a scaling factor of 10,000 and log-transformed (base 2), and the top 2,500 variable genes were scaled via the ScaleData function. Normalization returned 2 gene-cell matrices: one in log scale, and the other the adjusted gene-cell count. Principal component analysis (PCA) was performed with 30 principal components via the RunPCA function, followed by Harmony analysis to normalize the PCA embeddings across samples. We also used 15 as the number of dimensions for the FindNeighbors function. We ran the FindClusters function (resolution = 0.6) to identify clusters of cells and the RunUMAP function (reduction = “harmony,” dims = 1:20) for reduction to 2 dimensions for visualization purposes. The assignment of each cluster to a specific cell type was done with the SingleR package using the ImmGenData reference dataset. Differentially expressed genes between 2 groups were identified using the FindMarkers function with the default Wilcoxon’s rank-sum test, and genes with adjusted *P* value less than 0.05 were considered differentially expressed. Differentially expressed genes for each cell type were functionally annotated using the R package clusterProfiler (version 3.14.0). The mouse Kyoto Encyclopedia of Genes and Genomes (KEGG) and Gene Ontology databases were used to determine associations with particular biological processes, diseases, and molecular functions. The top pathways with an FDR-adjusted *P* value less than 5% were summarized in the results.

### Measurement of serum and urinary SNCA.

Serum and urinary SNCA levels were measured using the Mouse α-Synuclein ELISA Kit (Abcam, ab282865) per the manufacturer’s protocol. Urinary SNCA levels were normalized to creatinine.

### Quantification of albuminuria.

Urinary albumin was quantified using the Mouse Albumin ELISA kit (Bethyl Laboratories, NC0096469), according to the manufacturer’s protocol. Data were normalized to urine creatinine.

### BMDM cell culture.

Bone marrow–derived macrophages (BMDMs) were isolated from WT mice as previously described (74). BMDMs were cultured for 5 days in DMEM (Corning, 10-013-CV) with 15% FBS (R&D Systems, S11150), 1% penicillin-streptomycin (Gibco, 15140122), 1% MEM non-essential amino acids (Gibco, 11140050), and 0.3 μg/mL M-CSF (Miltenyi Biotec, 130-101-704). On day 6, the culture medium was replaced with DMEM containing 1% FBS for 2 hours, followed by treatment with either vehicle, mouse recombinant monomeric SNCA protein monomers (Proteos, RP-011), or fibrils at a dose of 1 μM (StressMarq, SPR-324). Dose of SNCA was established per previous reports (59). Cells were then washed with PBS and collected for RNA or protein analysis. Presence of LPS mouse recombinant SNCA monomers and fibrils was ruled out using the Pierce chromogenic endotoxin quantification kit (Thermo Fisher Scientific, A39552) according to the manufacturer’s instructions, where values were determined to be less than 0.01 EU/mL. Fibrils were sonicated at 4°C in a water bath sonicator for a total of 15 minutes, using a cycle of 3 seconds on and 2 seconds off. To assess the effects of heat and enzymatic digestion on SNCA, 1 μM of monomers were either boiled overnight at 100°C or treated with proteinase K (1 mg/mL) at 37°C overnight before addition to BMDM medium.

### HK-2 cell culture.

HK-2 cells, provided by Anupam Agarwal (UAB), were cultured following ATCC-recommended protocols. Cells were maintained in keratinocyte-SFM medium supplemented with epidermal growth factor, bovine pituitary extract (Gibco, 10724-011), and 1% (vol/vol) penicillin-streptomycin. Cell culture plates were precoated with 50 μg/mL Collagen Type I (Corning, 354236) before seeding. Cultures were kept at 37°C in a humidified incubator with 5% CO_2_, and the medium was replaced every 48–72 hours. Cells were allowed to grow for 3–5 days until they reached approximately 70%–80% confluence. At this stage, HK-2 cells were detached using trypsin and either passaged at a 1:3 ratio or seeded into 6- or 12-well plates at the desired density for experiments.

### Live-cell imaging for SNCA uptake.

For the uptake assay, BMDMs were plated on 35 mm MatTek live imaging dishes and were treated with 0.1 μM of Alexa Fluor 488–labeled (A488-labeled) SNCA monomers (StressMarq, SPR-517B-A488). Following A488-labeled SNCA treatment, extracellularly bound SNCA was quenched using trypan blue. Both A488 and Alexa Fluor 555 channels were used for imaging. A488 was used to visualize SNCA internalization and Alexa Fluor 555 to detect trypan blue binding to extracellular proteins. The internalization of A488-labeled SNCA was then assessed via live-cell imaging, as previously described (75).

### BODIPY (lipid peroxidation sensor) assay.

BMDMs were treated with either vehicle or monomeric SNCA (Proteos, RP-011) for 16 hours and incubated with 2 μM BODIPY 581/591 C11 (Invitrogen, D3922) for 30 minutes at 37°C in 5% CO_2_. Cells were washed with cold PBS to remove excess dye and resuspended in flow cytometry buffer (PBS containing 2% FBS and 0.1% sodium azide). Flow cytometry was performed using the Cytek Northern Lights instrument and analyzed using FlowJo software.

### Cellular ROS assay.

BMDMs were treated with either vehicle or SNCA monomers (Proteos, RP-011) for 16 hours and incubated with 10 μM DCFDA (Abcam, ab113851) for 30 minutes at 37°C in 5% CO_2_. Excess dye was removed via PBS washes, followed by fluorescent microscopy and flow cytometry analysis.

### Human samples.

Remnant human kidney biopsies were obtained through the tissue resource available from the UAB-UCSD O’Brien Center for AKI Research. These samples were collected, deidentified, archived together, and analyzed according to protocols approved by the Institutional Review Board of the UAB. Biopsy samples for this study were selected based on the diagnosis of ACR, AIN, or TBM, according to pathologists’ diagnoses and comments. Diagnosis of TBM was also confirmed by electron microscopy, and thickness measurement values used to establish the diagnosis of TBM were 337 ± 75 nm for females and 355 ± 75 nm for males (Supplemental Figure 11). The 3 studied groups, TBM, AIN, and ACR, were each represented by kidney biopsies from 6 patients. Patient characteristics are presented in Supplemental Table 3. Serum creatinine of 1 patient in the TBM and 1 patient in the AIN group was not available for analysis.

### Interpretation of gene expression data using publicly available transcriptomic platforms.

All data sources were contacted via email correspondence and provided explicit consent for the use of their datasets and the generation of figures for this study. Each source requested appropriate citation of their work, which has been duly incorporated in the manuscript. We used 3 publicly available resources for transcriptomic analysis: (a) The Kidney Precision Medicine Project (https://www.kpmp.org), accessed between September 2024 and February 2025, funded by the National Institute of Diabetes and Digestive and Kidney Diseases (grants U01DK133081, U01DK133091, U01DK133092, U01DK133093, U01DK133095, U01DK133097, U01DK114866, U01DK114908, U01DK133090, U01DK133113, U01DK133766, U01DK133768, U01DK114907, U01DK114920, U01DK114923, U01DK114933, U24DK114886, UH3DK114926, UH3DK114861, UH3DK114915, and UH3DK114937). Specifically, Supplemental Figure 9 and Supplemental Tables 1 and 2 were generated via accessing of this platform. (b) The Kidney Interactive Transcriptomics platform (http://humphreyslab.com/SingleCell/), developed by Benjamin Humphreys. The generated results in this study used a data source that was previously published (76, 77). Figure 7, K and L, and Supplemental Figure 10 were generated using this platform. (c) The DICE database website (https://dice-database.org) was used to generate data presented in Supplemental Figure 3.

### Statistics.

Statistical analyses were performed using appropriate methods based on the study design. Experiments involving 2 groups were analyzed by 2-tailed, unpaired Student’s *t* test. For analyses involving 3 or more independent groups, a 1-way ANOVA with Tukey’s or Dunnett’s post hoc correction was applied. To compare 2 groups across 2 conditions, a 2-way ANOVA with Tukey’s or Šidák’s correction for multiple comparisons was conducted. All data are expressed as mean ± SEM. Statistical tests were 2-sided and were performed using GraphPad Prism v10, with details provided in the figure legends. Statistical significance was defined as follows: not significant (NS), *P* > 0.05; *P < 0.05; **P < 0.01; ***P < 0.001.

### Study approval.

All mouse experiments were performed in accordance with NIH guidelines for the use and care of live animals and were reviewed and approved by the UAB’s Institutional Animal Care and Use Committee. Human tissue samples were procured and approved by the UAB’s Institutional Review Board.

### Data availability.

The sequencing data for this study were deposited in the Gene Expression Omnibus and are publicly available as of the date of publication under accession numbers GSE292295 and GSE291562. Values for all data points in graphs are reported in the Supporting Data Values file.

## Author contributions

TC designed and performed experiments, collected and analyzed data, formulated publishable figures, and wrote the manuscript. SM performed experiments, collected and analyzed data, and assisted in manuscript preparation. KC, MM, and BJ performed experimentation. YZ conducted all analyses for scRNA-seq. LVD conceived the project and provided resources. LF, RP, FR, and LP participated in experimentation. GB, JB, and CF conceived the project, conducted investigations, and reviewed and edited the manuscript. AZ conceived the project, designed the study, supervised experimental work, analyzed data, generated figures, wrote the manuscript, and secured funding.

## Conflict of interest

CF has served as a consultant for Bayer and Calico Labs. CF is an inventor on two pending patents (PCT/US2019/049211; PCT/US19/49161) and has a patent on FGFR inhibition (European Patent 2723391). CF is the cofounder and CSO of a startup biotech company (Alpha Young LLC).

## Funding support

This work is the result of NIH funding, in whole or in part, and is subject to the NIH Public Access Policy. Through acceptance of this federal funding, the NIH has been given a right to make the work publicly available in PubMed Central.

- NIH grant DK134402 (to AZ).
- UAB-UCSD O’Brien Center for Acute Kidney Injury Research (NIH U54 DK137307).
- Project 2025-1.2.1-HU-RIZONT-2025-00080 implemented with support provided by the Ministry of Culture and Innovation of Hungary from the National Research, Development and Innovation Fund, financed under the 2025-1.2.1-HU-RIZONT funding scheme (to AZ, JB, and GB).
- American Heart Association (AHA) Postdoctoral Fellowship Grant 26POST1559305 (to TC).

## Supplementary Material

Supplemental data

Unedited blot and gel images

Supporting data values

## Version 1 03/10/2026

In-Press Preview

## Version 2 04/22/2026

Electronic publication
