# Supplementary material for: Macrophage ferritin heavy chain/α-synuclein regulatory axis modulates ferroptosis during kidney injury
Source: JCI Insight. 2026 Mar 10;11(8):e196521. doi: 10.1172/jci.insight.196521 (PMC13135404; doi:10.1172/jci.insight.196521)
Supplement: Supplemental data [file jciinsight-11-196521-s094.pdf]

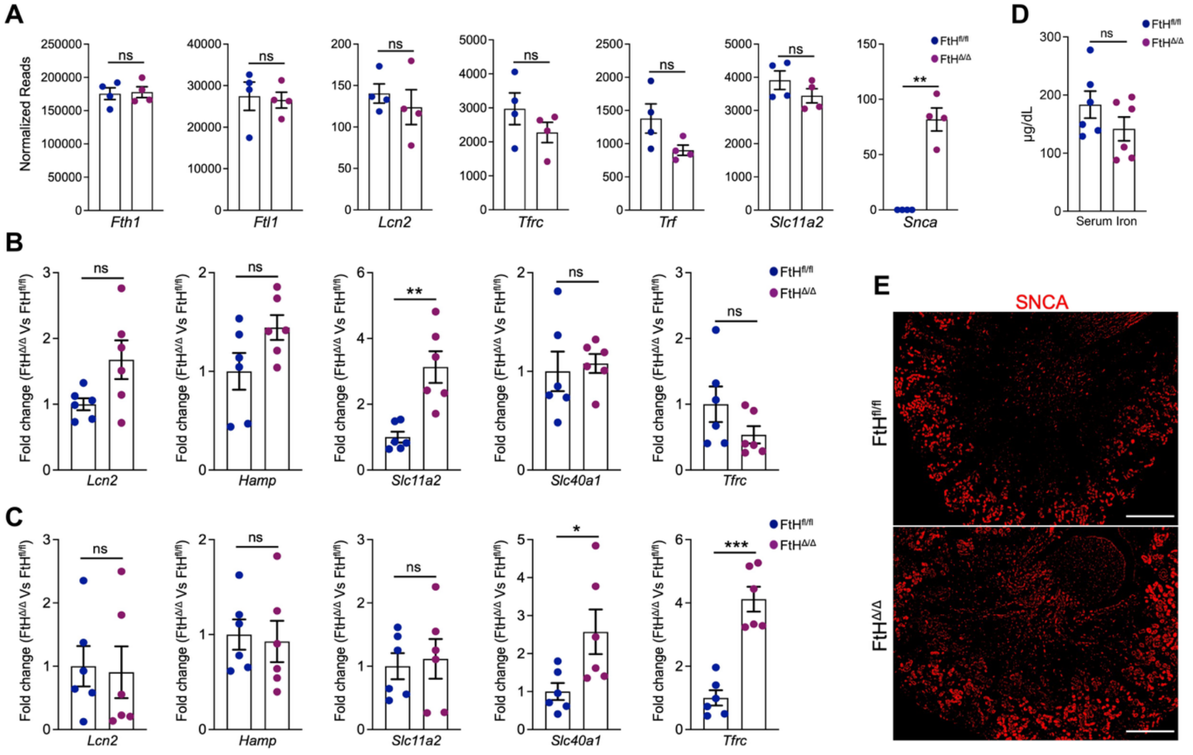

Figure S1. (A) Bulk RNA-sequencing data of FtH$^{fl/fl}$ and FtH$^{\Delta/\Delta}$ kidneys under baseline conditions highlighting genes that encode key iron regulatory proteins. Hepcidin (Hamp) levels were undetectable. (n = 4). Normalized count data of signature genes are plotted. ns = not significant, **P < 0.01. (B, C) FtH$^{fl/fl}$ and FtH$^{\Delta/\Delta}$ livers (B) and spleens (C) were harvested under baseline conditions and mRNA expression levels of *Lcn2* (Ngal), *Hamp* (hepcidin), *Slc11a2* (DMT1), *Slc40a1* (Fpn), and *Tfrc* (transferrin receptor 1) were analyzed using qRT-PCR. Data are normalized to *Gapdh* and represented as fold change, FtH$^{fl/fl}$ Vs. FtH$^{\Delta/\Delta}$. ns = not significant, *P < 0.05, **P < 0.01, ***P < 0.001. (D) Serum iron concentrations in FtH$^{fl/fl}$ and FtH$^{\Delta/\Delta}$ under quiescent conditions were measured and reported in µg/dL. ns = not significant. (E) Immunofluorescence staining of SNCA in kidneys of FtH$^{fl/fl}$ and FtH$^{\Delta/\Delta}$ mice under baseline conditions. Scale bar = 50 µm.

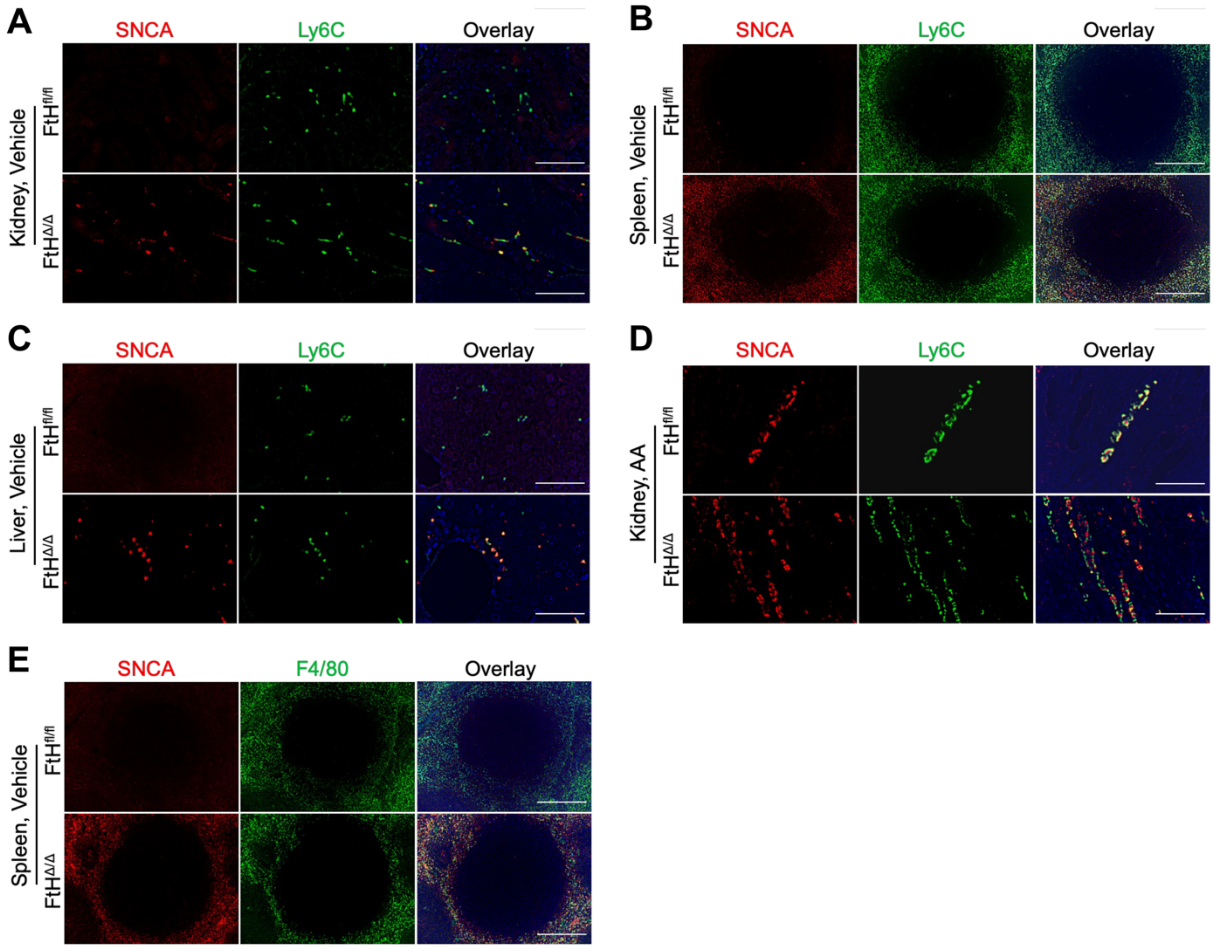

Figure S2. (A-C) Immunofluorescence staining of SNCA and Ly6C in the kidney, spleen, and liver of $FtH^{fl/fl}$ and $FtH^{\Delta/\Delta}$ mice under baseline conditions. (D) Upregulation of SNCA in $Ly6C^{+}$ cells following AA-induced injury in both genotypes. Scale bars: kidney= 25 μm, spleen= 200 μm, liver= 25 μm. (E) Immunofluorescence assay illustrating co-expression of SNCA and F4/80 in spleen of $FtH^{\Delta/\Delta}$ mice. Scale bar = 200 μm.

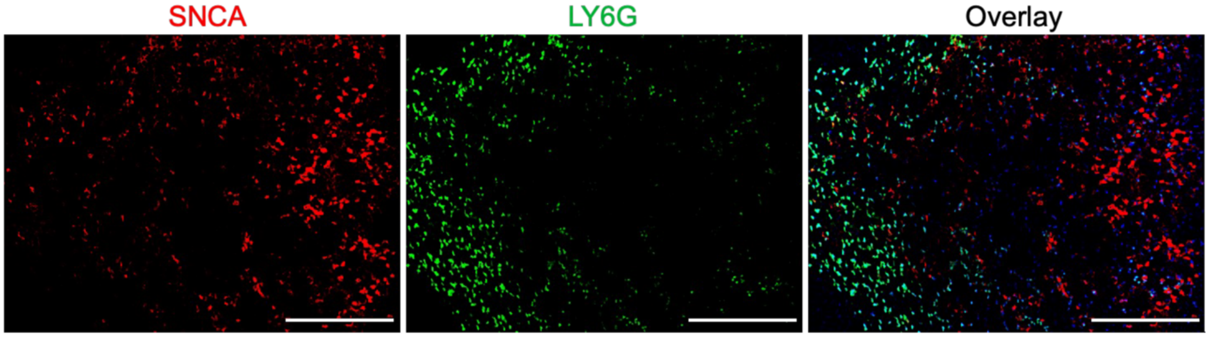

Figure S3. Immunofluorescence assays were performed using anti-SNCA and anti-LY6G antibodies to determine the pattern of expression of these proteins in spleen of $FtH^{\Delta/\Delta}$ mice. As illustrated neutrophils are not the primary source of SNCA expression in $FtH^{\Delta/\Delta}$ spleens. Scale bar = 100 μm.

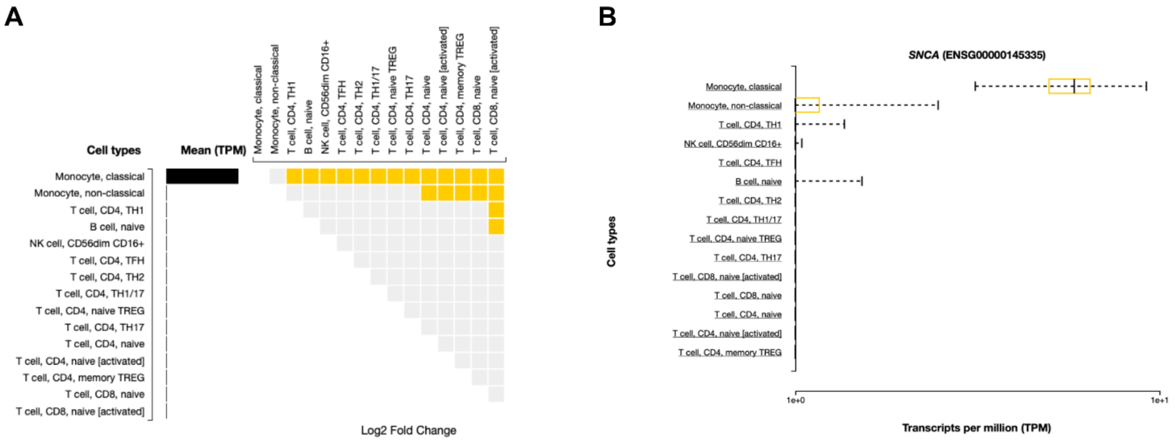

Figure S4. Expression of SNCA in leukocytes as determined by (A) log2 fold change, and (B) transcripts per million reveals monocytes as the major cellular source of expression. Data was generated via accessing publicly available transcriptome analysis platform. (https://dice-database.org)

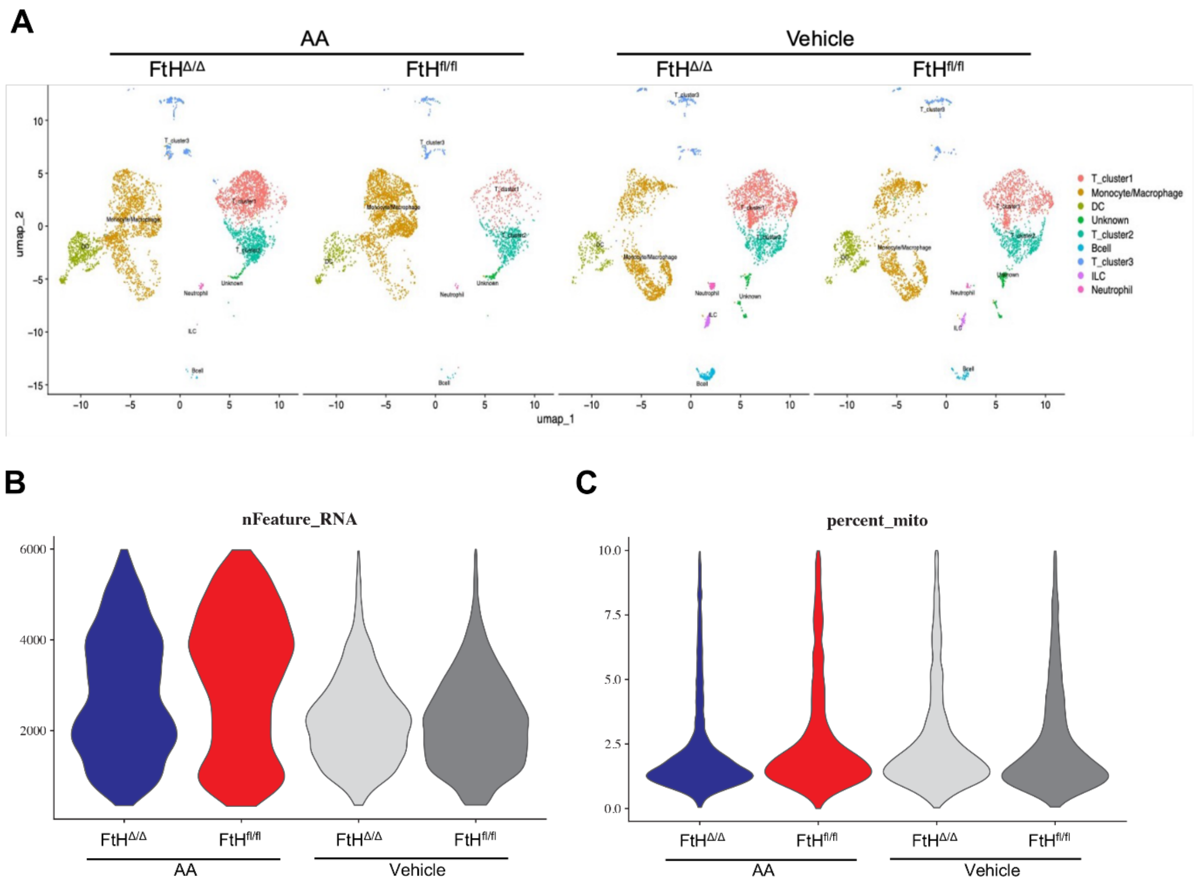

Figure S5. (A) UMAP projection of isolated CD45$^+$ (leukocytes) from kidneys of both genotypes, treated with vehicle or AA, passing rigid quality control filtering and after dataset integration, yielding 9 distinct cell clusters (n= 1 per genotype and treatment condition). (B, C) Violin plots showing (B) the number of informative genes per single cell, and (C) the percentage of mitochondrial genes per single cell, all split by batches.

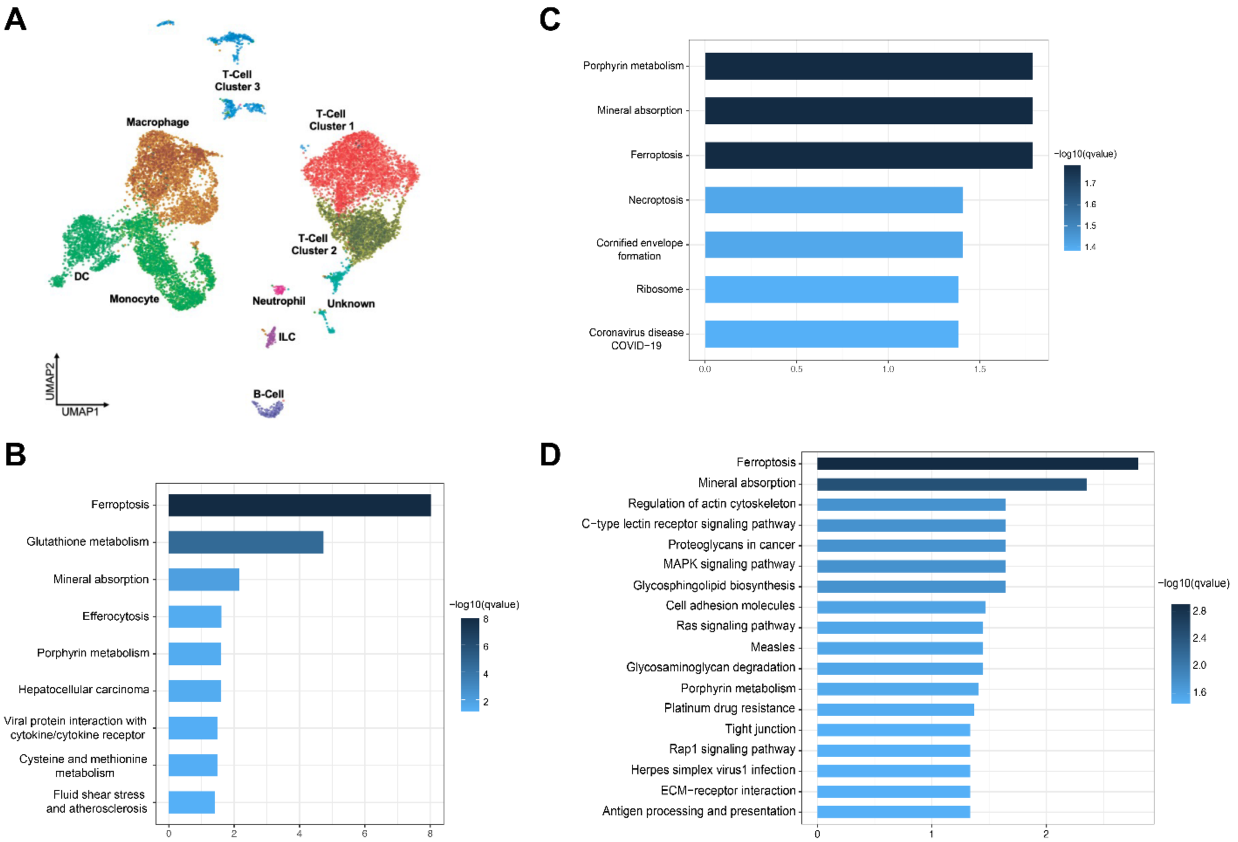

Figure S6. (A) Uniform manifold approximation and projection (UMAP) plot showing the clustering of immune cells (CD45$^+$) in the kidney based on single-cell RNA sequencing. The analysis identifies ten distinct clusters, with contaminating kidney cells and clusters representing less than 1% removed. Specifically, monocytes and MΦ were clustered as distinct populations to enable independent assessment of pathway enrichment under each experimental condition. (B) Pathway enrichment analysis of differentially expressed genes in FtH deficient monocytes versus wildtype monocytes under vehicle-treated conditions. (C) Pathway enrichment analysis of differentially expressed genes in FtH deficient monocytes versus wildtype monocytes following AA administration. (D) Pathway enrichment analysis of differentially expressed genes in MΦ derived from FtH$^{\Delta/\Delta}$ kidneys versus MΦ derived from FtH$^{fl/fl}$ kidneys following AA administration.

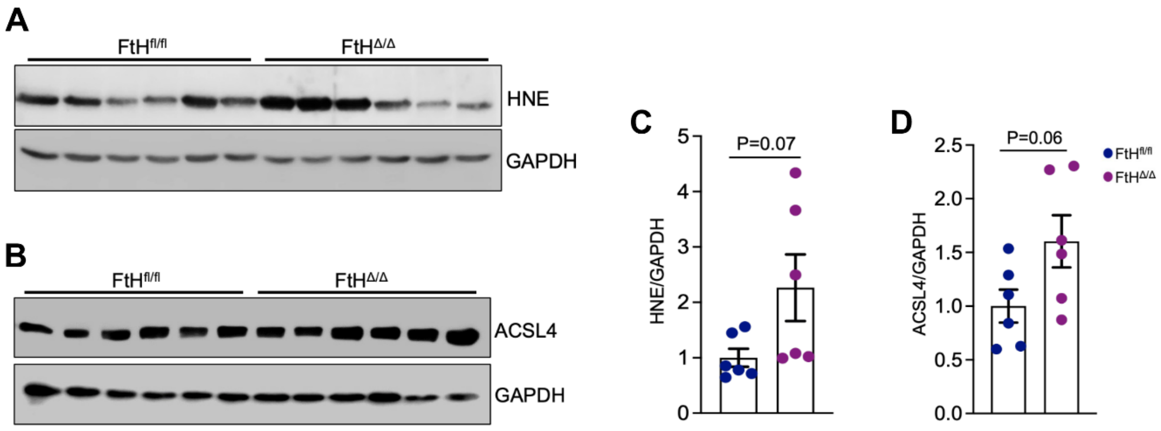

Figure S7. (A, B) FtH$^{fl/fl}$ and FtH$^{\Delta/\Delta}$ kidneys were harvested under baseline conditions and protein expression of (A) HNE, and (B) ACSL4 were examined via Western blotting. (C, D) Densitometric analysis indicates an increasing trend for both (C) HNE, and (D) ACSL4 in baseline FtH$^{\Delta/\Delta}$ kidneys; however, these results did not achieve statistical significance.

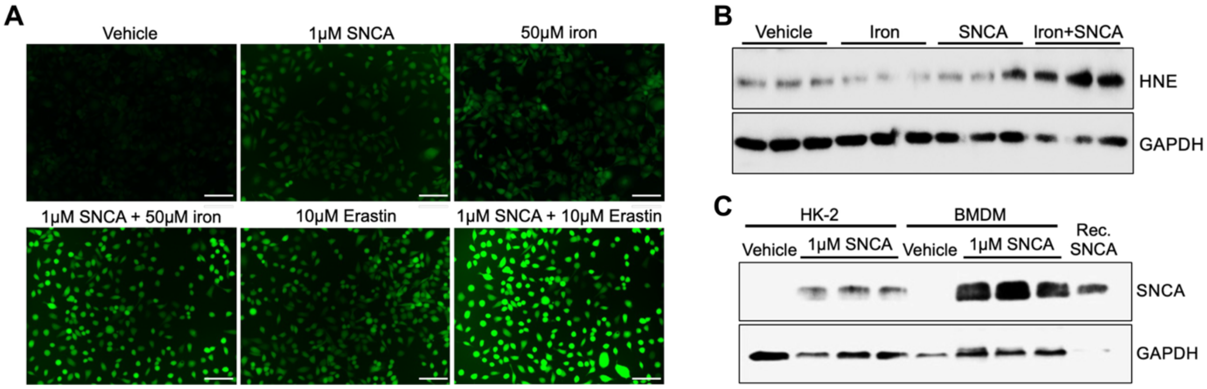

Figure S8. Representative images demonstrating intracellular ROS using DCFDA in HK-2 cells. DCFDA staining was performed at 16 hours post treatment with SNCA alone, iron alone, SNCA and iron, Erastin alone, Erastin and SNCA, at denoted concentrations. Scale bar = 200 μm. (B) HK-2 cells were treated with vehicle, 50 μM iron alone, 1 μM SNCA alone, and combination of 50 μM iron and 1 μM SNCA for 16 hours. Cell lysates were used to detect HNE levels. GAPDH was used as loading control. (C) To detect SNCA levels western blotting was performed using protein lysates of HK-2 cells and BMDMs subjected to 1 μM SNCA for 16 hours. GAPDH was used as loading control.

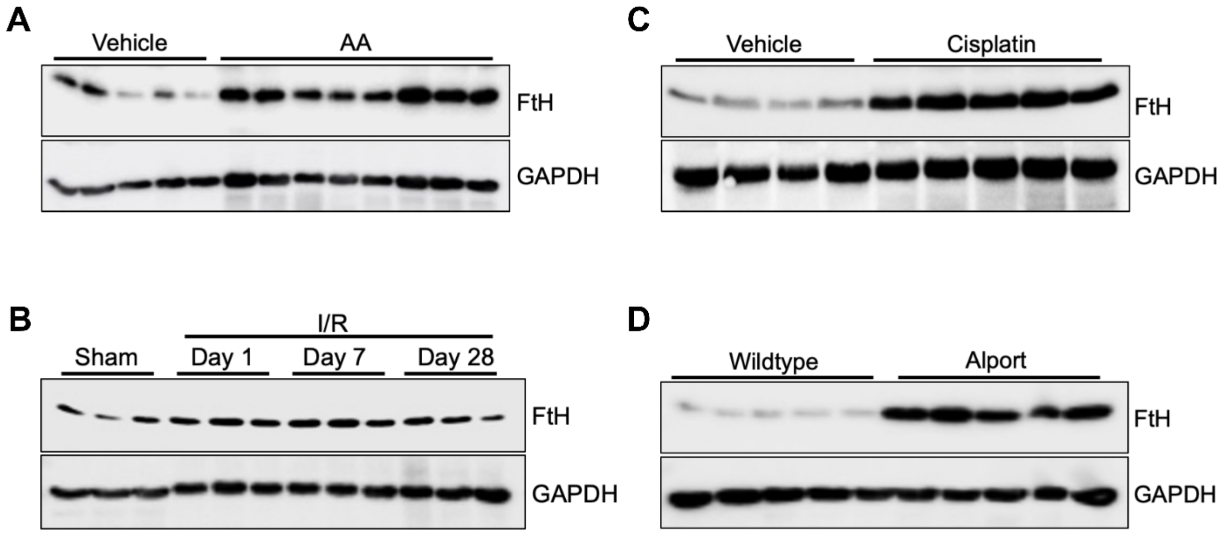

Figure S9. (A-D) Western blot analysis of FtH in kidney lysates from four different mouse models of kidney disease: (A) Wildtype mice were treated with vehicle or aristolochic acid (AA), and kidney lysates were collected six weeks post-AA administration. (B) Wildtype mice subjected to 20 minutes of ischemia-reperfusion (I/R) injury. Kidney samples were collected at indicated time-points. (C) Protein expression of FtH in wildtype kidneys treated with cisplatin as described in methods. (D) FtH expression in kidneys from wildtype ($Col4a3^{+/+}$) and Alport ($Col4a3^{-/-}$) mice at twelve weeks of age. GAPDH was used as loading control.

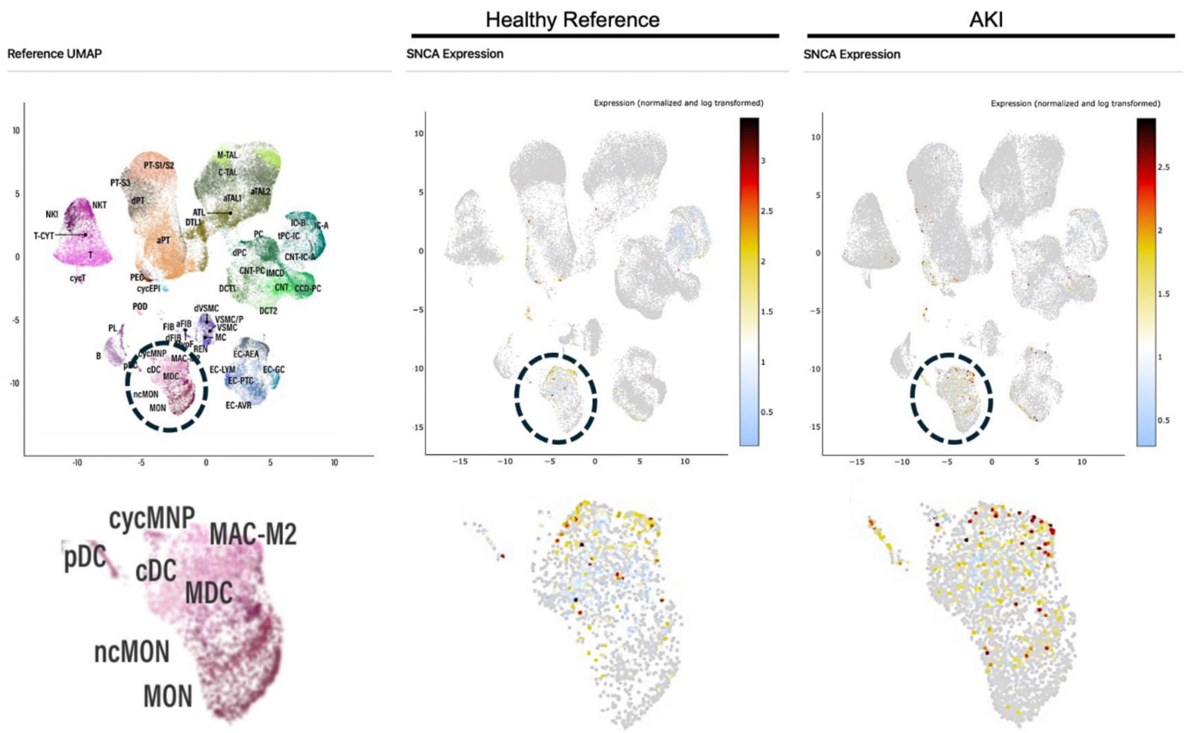

Figure S10. Reference UMAP, the left panel, shows different annotated cell types. The black dash sequesters cells of monocytic lineage subpopulation of leukocytes and was isolated for better visualization. The middle panel shows expression levels of in healthy kidneys and the right panel demonstrates enrichment of SNCA during AKI. Data was generated via accessing kidney precision medicine project website. (https://www.kpmp.org)

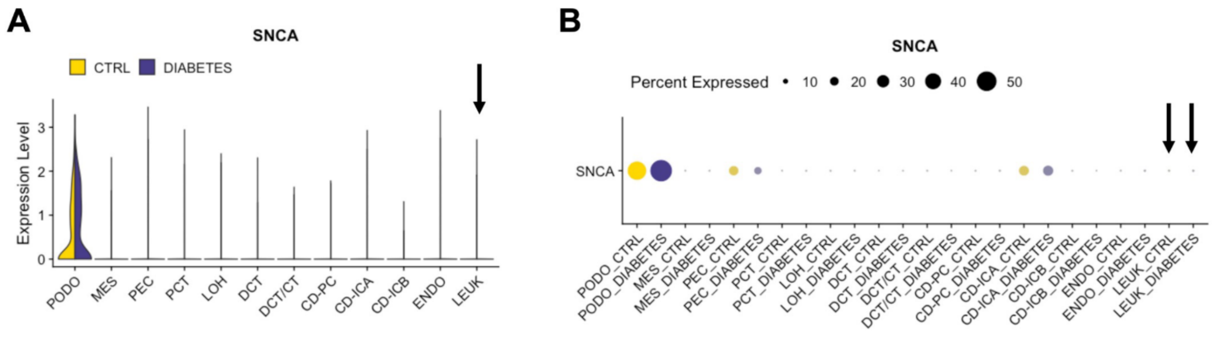

Figure S11. (A) violin plot of Sc-RNAseq demonstrates enrichment of SNCA in control and diabetic kidneys that is almost exclusively seen in podocytes. Arrow indicates expression of SNCA in isolated leukocytes. (B) Dotplot analysis confirms lack of enrichment of SNCA in leukocytes as indicated by arrows. Data was generated via accessing kidney interactive transcriptomics platform developed by Dr. Humphreys. (https://humphreyslab.com/SingleCell/)

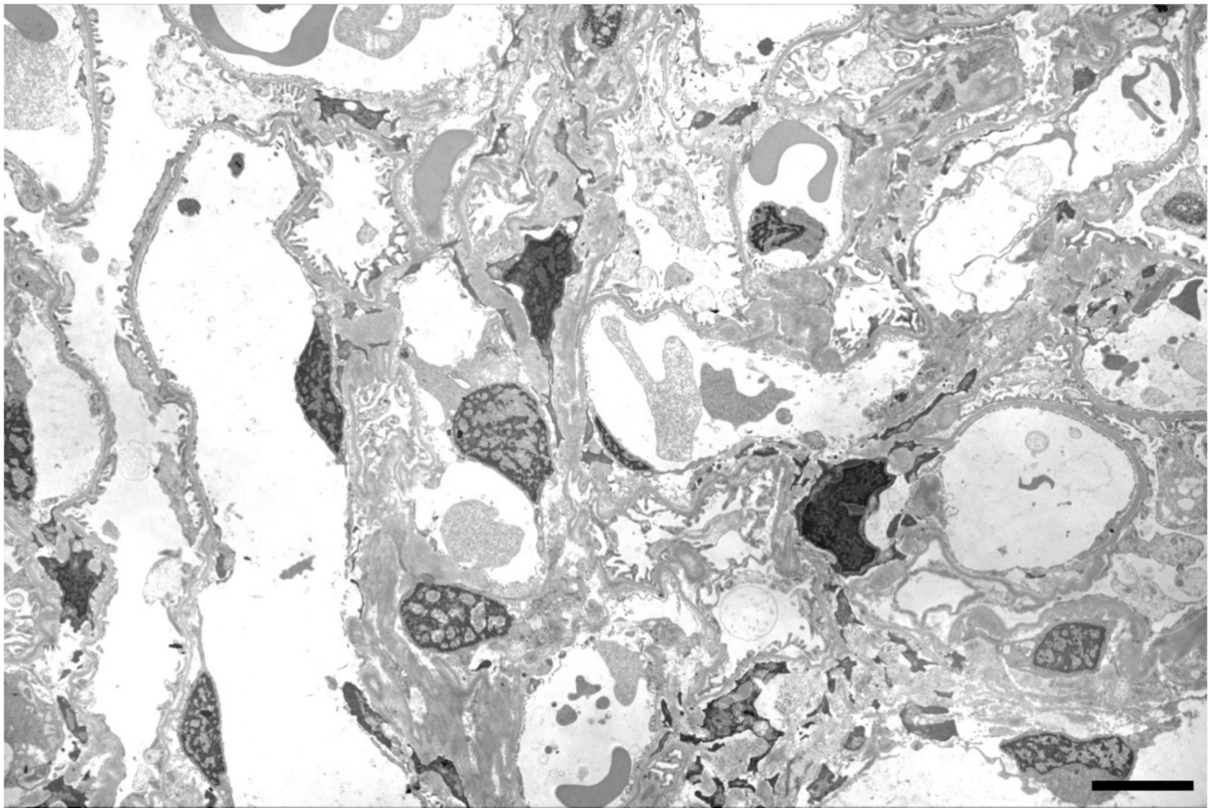

Figure S12. Representative images of kidney biopsies with an isolated diagnosis of TBM demonstrate thinning of the basement membrane. Scale bar = 5 μm.

Supplemental Table 1. Cluster of cells that significantly express *Snca* mRNA in kidneys of healthy individuals

cluster Abbrev	cluster Name	cell Count	Mean Exp	pct Cells Expressing	fold Change	p Val	p Val Adj
MDC	Monocyte-derived Cell	1017	0.68	37.2	3.21	4.32E-135	1.39E-130
POD	Podocyte	201	1.95	52.2	4.63	5.32E-133	1.71E-128
IC-A	Intercalated Cell Type A	1980	0.322	18.6	2.07	1.16E-52	3.72E-48
MAC-M2	M2-Macrophage	465	0.852	19.6	3.43	8.51E-42	2.74E-37
PEC	Parietal Epithelial Cell	242	0.565	21.5	2.74	2.88E-26	9.24E-22
cDC	Classical Dendritic Cell	298	0.647	18.1	2.95	8.44E-23	2.71E-18
pDC	Plasmacytoid Dendritic Cell	36	0.666	38.9	2.99	2.39E-22	7.69E-18
CNT-IC-A	Connecting Tubule Intercalated Cell Type A	712	0.171	14	1	3.96E-18	1.27E-13
cycMNP	Mononuclear Phagocyte (*cycling*[2])	14	0.59	42.9	2.92	1.39E-13	4.46E-09
MON	Monocyte	459	0.223	10.5	1.39	1.06E-07	0.00339

Supplemental Table 2. Cluster of cells that significantly express *Snca* mRNA in kidneys of patients with AKI

cluster Abbrev	cluster Name	cell Count	mean Exp	pct Cells Expressing	fold Change	p Val	p Val Adj
PEC	Parietal Epithelial Cell	178	0.858	28.7	3.88	5.69E-73	1.83E-68
MAC-M2	M2-Macrophage	794	0.588	21.2	3.47	5.88E-70	1.89E-65
POD	Podocyte	57	1.99	47.4	5.08	7.86E-67	2.53E-62
cyc MNP	Mononuclear Phagocyte (*cycling*[2])	108	0.713	25.9	3.59	5.08E-42	1.63E-37
cDC	Classical Dendritic Cell	1012	0.339	12	2.61	8.18E-34	2.63E-29
pDC	Plasmacytoid Dendritic Cell	158	0.699	20.3	3.57	8.01E-30	2.57E-25
MDC	Monocyte-derived Cell	582	0.449	13.4	2.99	8.58E-29	2.76E-24

Supplemental Table 3. Characteristics of analyzed human kidney biopsy tissues

	**TBM (n=6)**	**AIN (n=6)**	**ACR (n=6)**
Age [years]	46±5.76	47±10.79	43±6.0
Gender: female [%]	100%	50%	66.6%
Max serum creatinine [mg/dL]	0.9±0.15	8.7±4.24	4.0±0.76

Supplemental Table 4. List of primary antibodies and dilutions used in Immunoblotting, Immunohistochemistry, and Immunofluorescence experiments

Primary Antibody	Source	Catalog Number	Dilution for Immunoblotting
Purified Mouse Anti-α-Synuclein	BD Biosciences	Cat #610787	1:1000
Anti-ACSL4 (FACL4) antibody [EPR8640]	Abcam	Cat #ab155282	1:2500
Anti-ferritin heavy chain Antibody (C6)	Santa Cruz Biotechnology	Cat #sc-517438	1:10,000 spleens, 1:1000 all other samples
Anti-Glyceraldehyde-3-Phosphate Dehydrogenase Antibody, clone 6C5	Millipore Sigma	Cat #MAB374	1:10,000
Anti-4 Hydroxynonenal antibody	Abcam	Cat #ab46545	1:2500
Ferritin Light Chain Polyclonal Antibody	Invitrogen	Cat #PA5-19059	1:1000
**Primary Antibody**	**Source**	**Catalog Number**	**Dilution for Immunohistochemistry**
α-Synuclein (D37A6) XP® Rabbit mAb	Cell Signaling Technology	Cat #4179S	1:200
Anti-4 Hydroxynonenal antibody	Abcam	Cat #ab46545	1:1000
**Primary Antibody**	**Source**	**Catalog Number**	**Dilution for Immunofluorescence**
α-Synuclein (D37A6) XP® Rabbit mAb	Cell Signaling Technology	Cat #4179S	1:200
CD11b Monoclonal Antibody (M1/70), Super Bright™ 600, eBioscience™	ThermoFisher Scientific	Cat #63011282	1:200
Alexa Fluor® 700 anti-mouse Ly-6G Antibody	BioLegend	Cat #127622	1:200
Alexa Fluor® 488 Conjugate. anti-mouse F4/80 (D4C8V) Rabbit Monoclonal Antibody	Cell Signaling Technology	Cat #27076	1:200
BD Pharmingen™ FITC Rat Anti-Mouse Ly-6C	BD Biosciences	Cat #553104	1:200

Supplemental Table 5. List of oligonucleotide sequences

q-PCR primer sequences			
**Species**	**Gene Name**		**Primer Sequence 5’-> 3’**
Mouse	*Gapdh*	FWD	ATCATCCCTGCATCCACT
Mouse	*Gapdh*	REV	ATCCACGACGGACACATT
Mouse	*Nfe2l2* (Nrf2)	FWD	CAGCATAGAGCAGGACATGGAG
Mouse	*Nfe2l2* (Nrf2)	REV	GAACAGCGGTAGTATCAGCCAG
Mouse	*Slc40a1* (ferroportin)	FWD	GTGGAGTACTTCTTGCTCTGG
Mouse	*Slc40a1* (ferroportin)	REV	CTGCTTCAGTTCTGACTCCTC
Mouse	*Slc7a11* (xCT)	FWD	GATTCATGTCCACAAGCACAC
Mouse	*Slc7a11* (xCT)	REV	GAGCATCACCATCGTCAGAG
Mouse	*Lcn2* (Ngal)	FWD	AACATTTGTTCCAAGCTCCAGGGC
Mouse	*Lcn2* (Ngal)	REV	CAAAGCGGGTGAAACGTTCCTTCA
Mouse	*Hamp* (hepcidin)	FWD	CAGCACCACCTATCTCCATCAAC
Mouse	*Hamp* (hepcidin)	REV	CAGATGGGGAAGTTGGTGTCTC
Mouse	*Slc11a2*(DMT-1)	FWD	TGTCACCGTCAGTATCCCA
Mouse	*Slc11a2* (DMT-1)	REV	ATGGCTGAGCCAATGACTTC
Mouse	*Tfrc* (Transferrin Receptor-1)	FWD	GGATTCATGAGTGGCTACCT
Mouse	*Tfrc* (Transferrin Receptor-1)	REV	CTGACTTGTCTGTCTCCTCC
**Genotyping primer sequences**			
Mouse	Cyclization recombinase (Cre)	FWD	GCCAGGCGTTTTCTGAGCATAC
Mouse	Cyclization recombinase (Cre)	REV	CACCATTGCCCCTGTTTCACTATC
Mouse	Ferritin heavy chain 1 (*FtH1*)	*FtH1* flox FWD	CCATCAACCGCCAGATCAAC
Mouse	Ferritin heavy chain 1 (*FtH1*)	*FtH1* flox REV	CGCCATACTCCAGGAGGAAC
