# Supplementary material for: Macrophage ferritin heavy chain/α-synuclein regulatory axis modulates ferroptosis during kidney injury
Source: JCI Insight. 2026 Mar 10;11(8):e196521. doi: 10.1172/jci.insight.196521 (PMC13135404; doi:10.1172/jci.insight.196521)

Full unedited blot for Figure 1E, Representative

SNCA

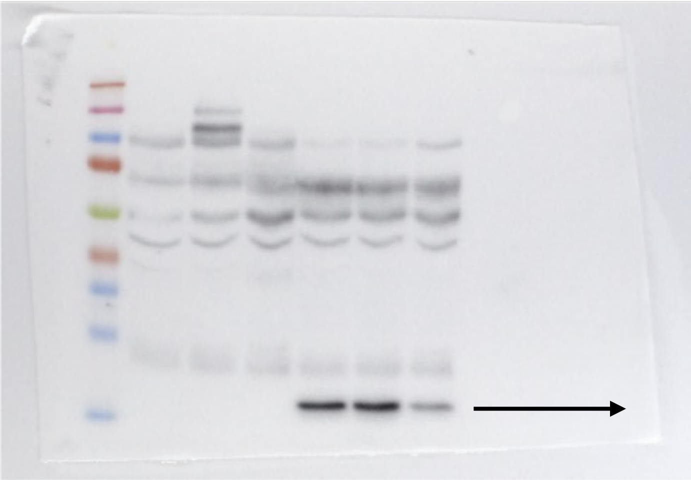

SNCA

GAPDH

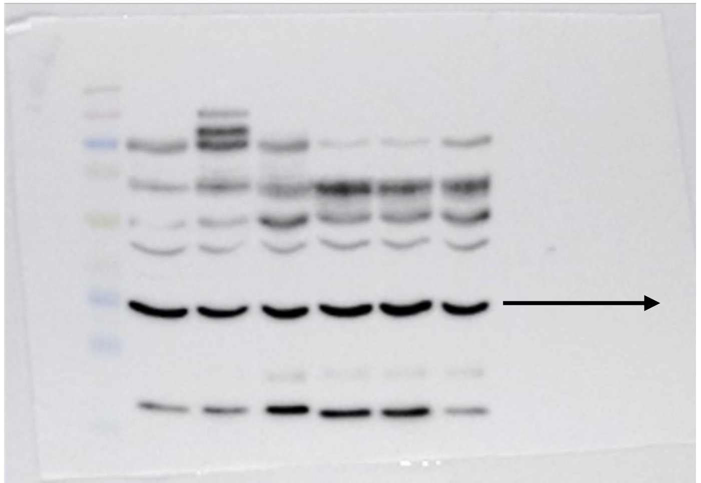

GAPDH

FtH

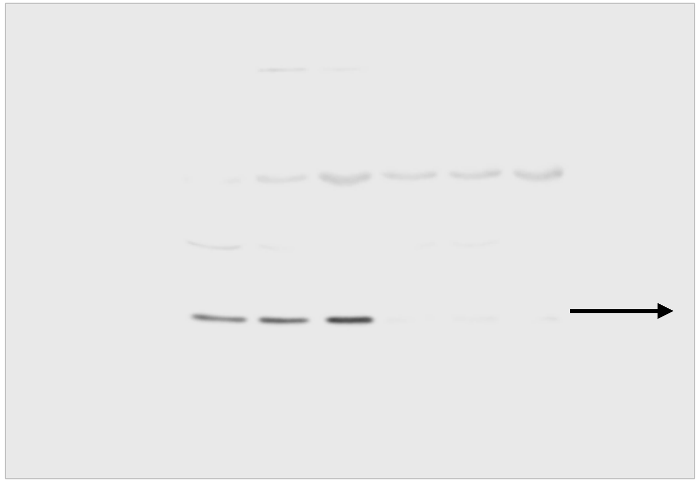

FtH

Figure 1E, used in densitometry

SNCA

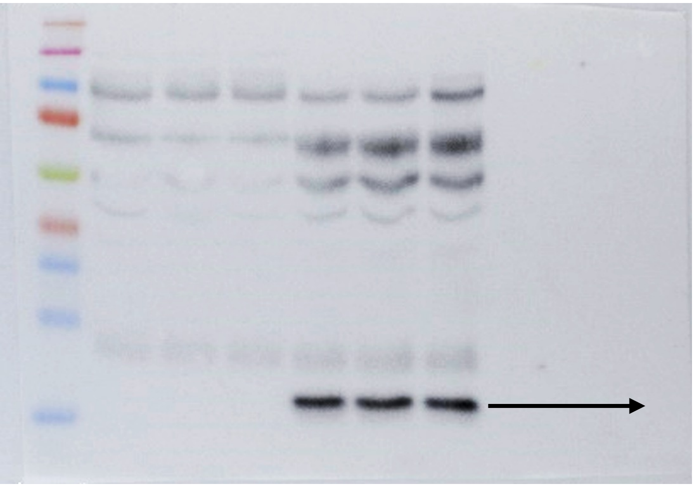

SNCA

GAPDH

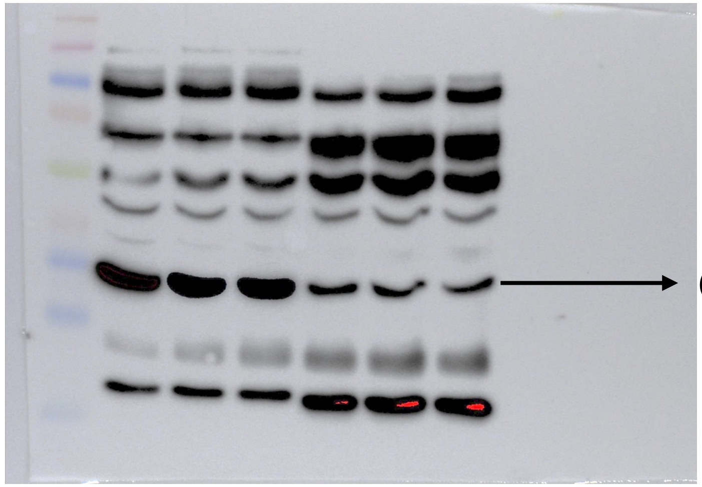

GAPDH

FtH

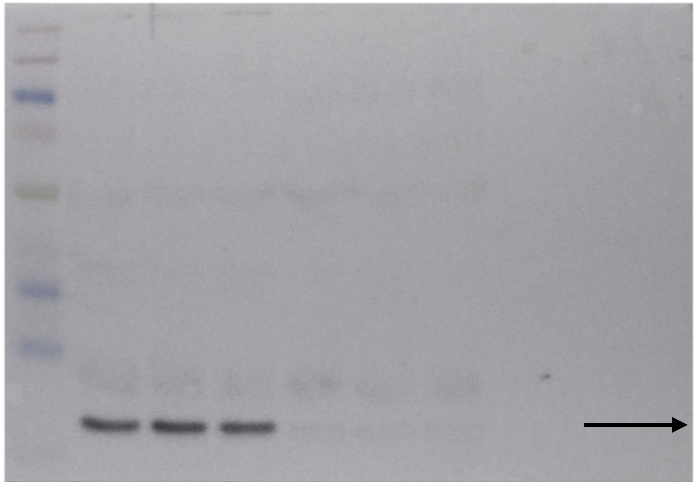

FtH

Full unedited blot for Figure 4 A

HNE

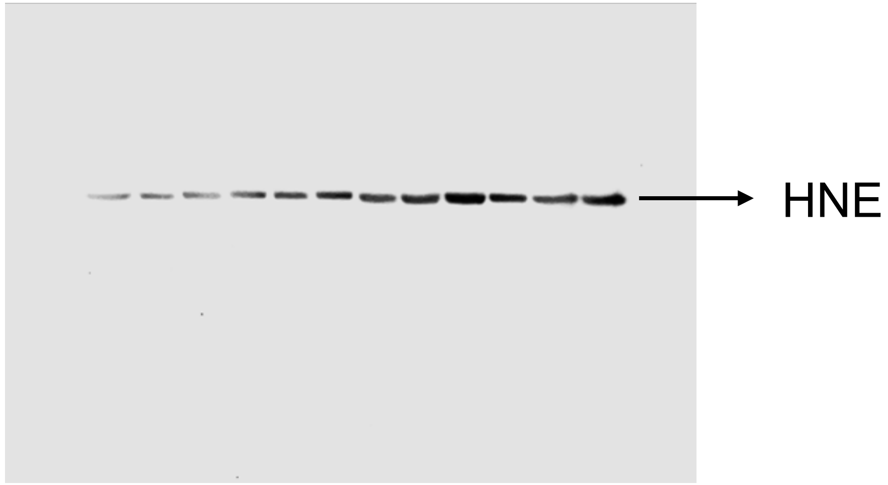

GAPDH

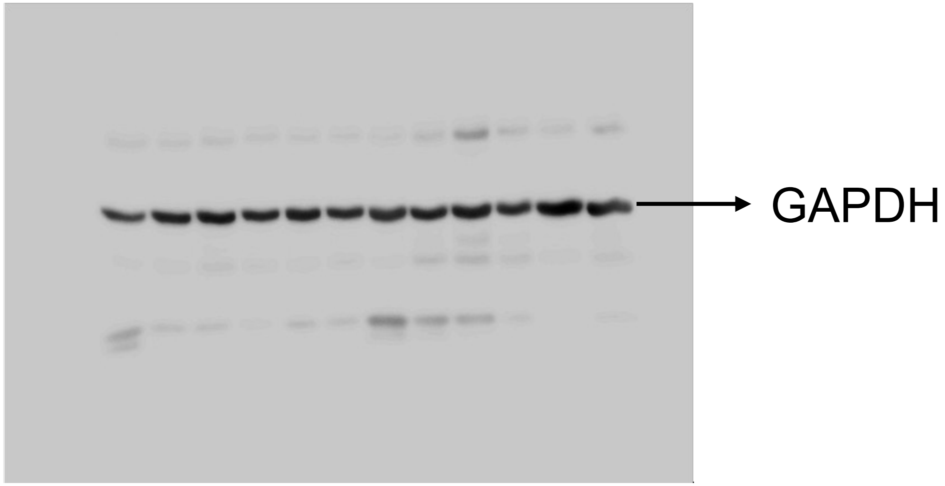

Full unedited blot for Figure 4B, Representative

ACSL4

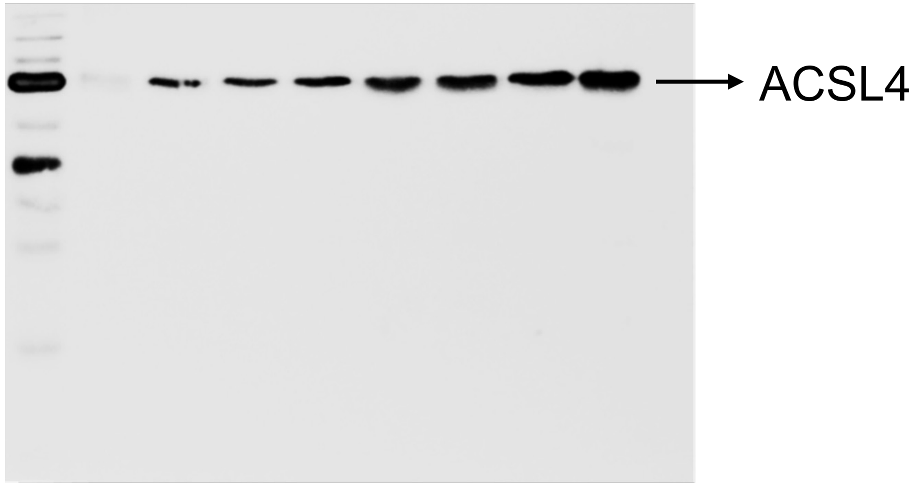

GAPDH

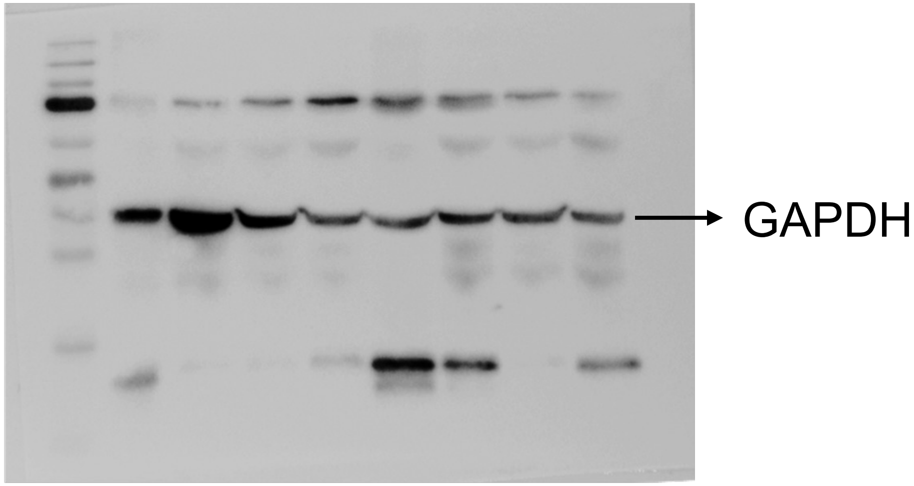

Full unedited blot for Figure 4B, used in densitometry

ACSL4

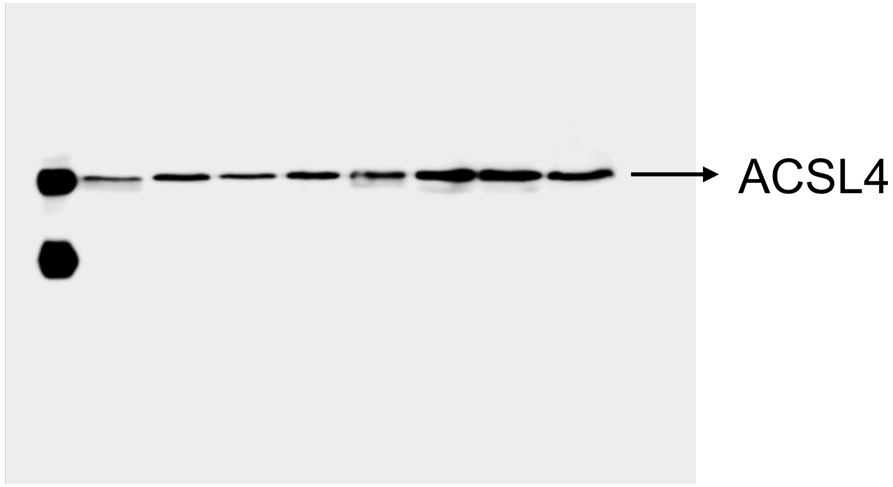

GAPDH

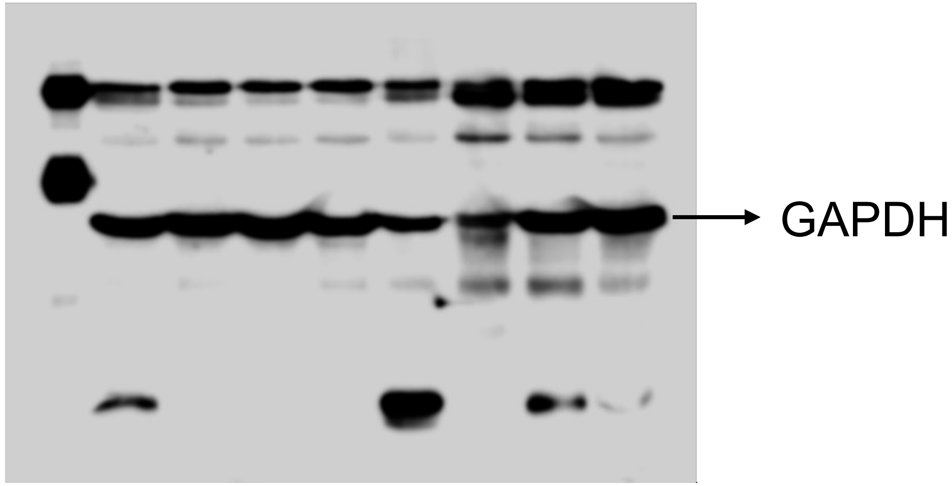

Full unedited blot for Figure 6A

SNCA

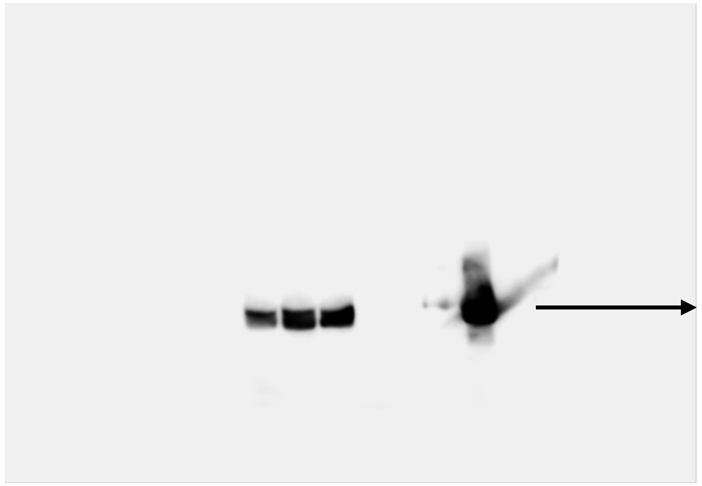

SNCA

GAPDH

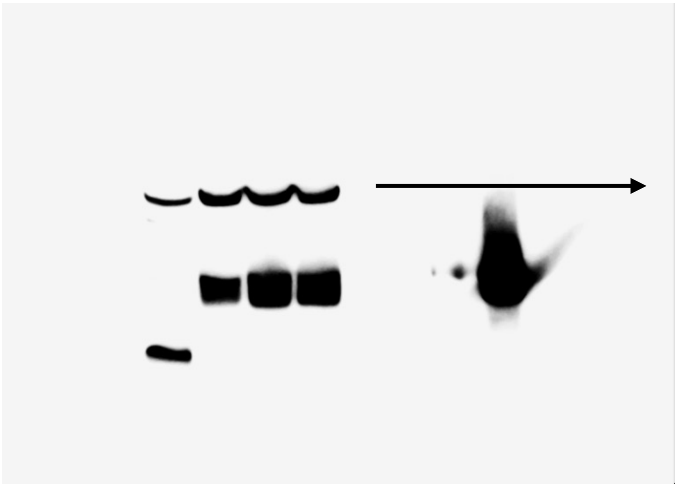

GAPDH

Full unedited blot for Figure 6C, Representative

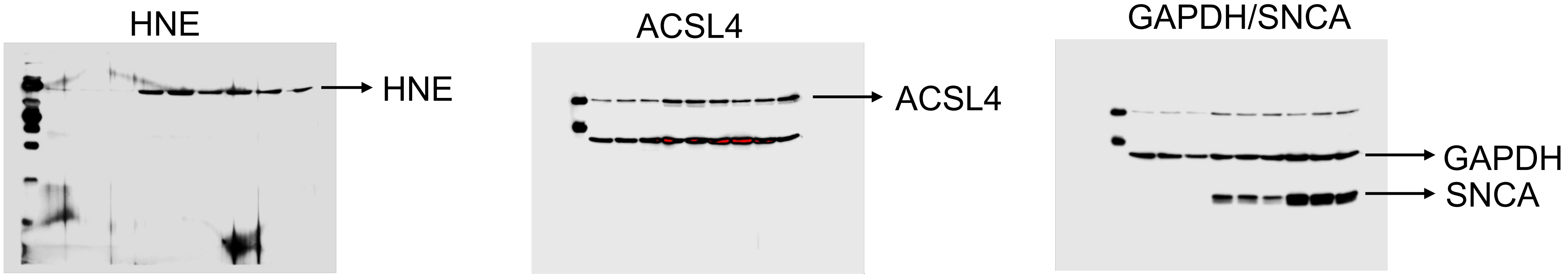

Full unedited blot for Figure 6C, used in densitometry

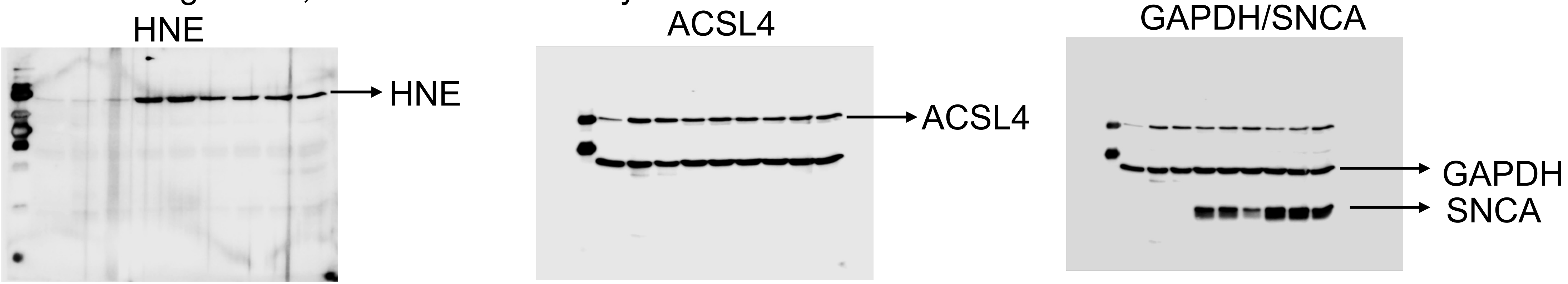

Full unedited blot for Figure 6C, used in densitometry

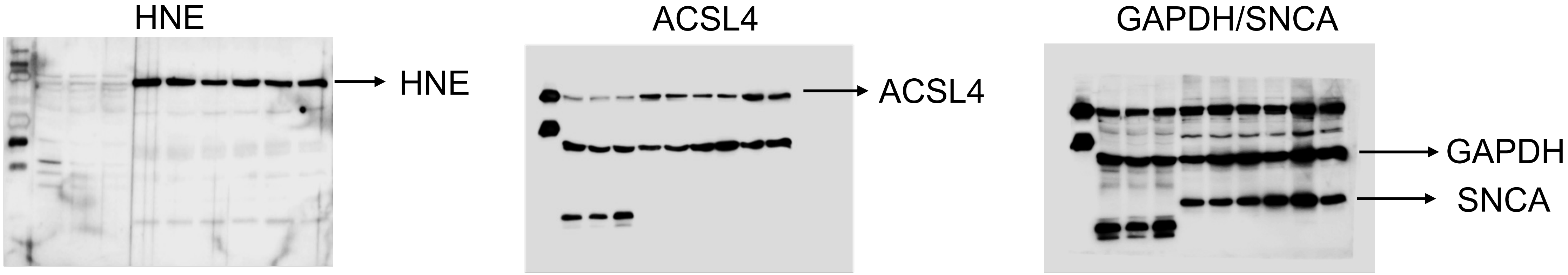

Full unedited blot for Figure 6O, Representative

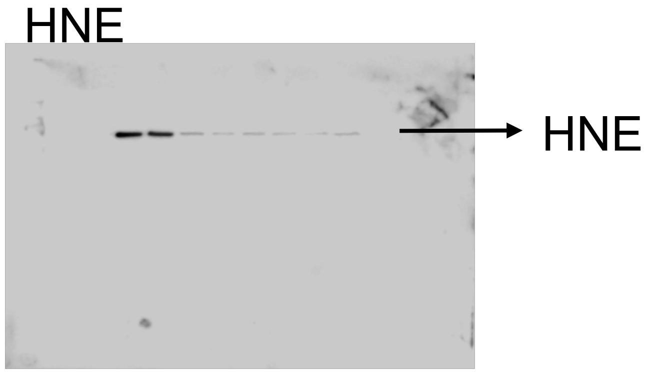

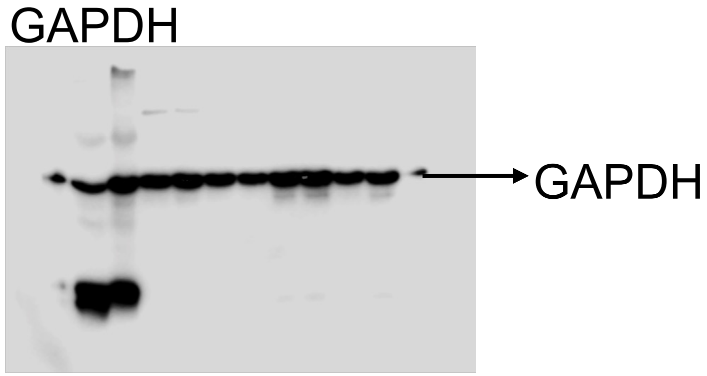

Full unedited blot for Figure 6O, used in densitometry

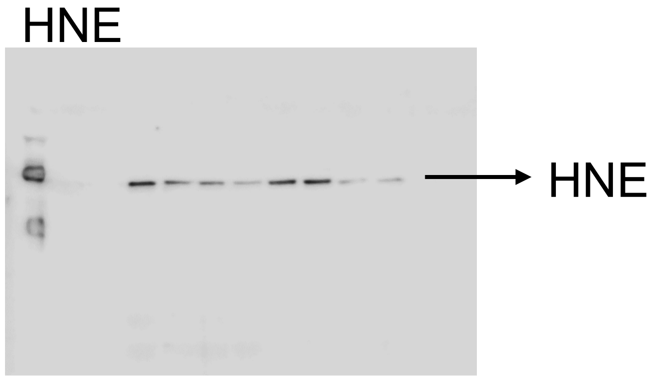

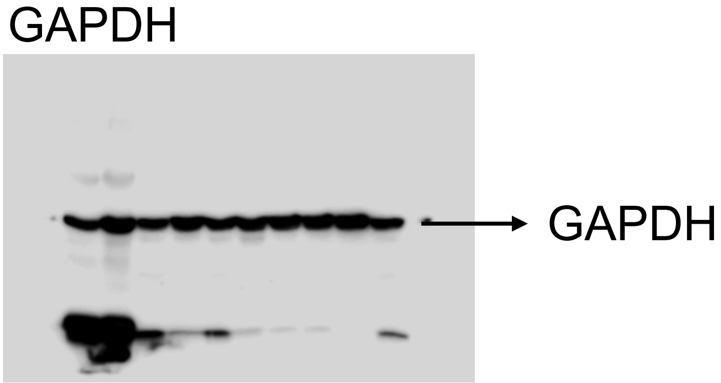

Full unedited blot for Figure 6O, used in densitometry

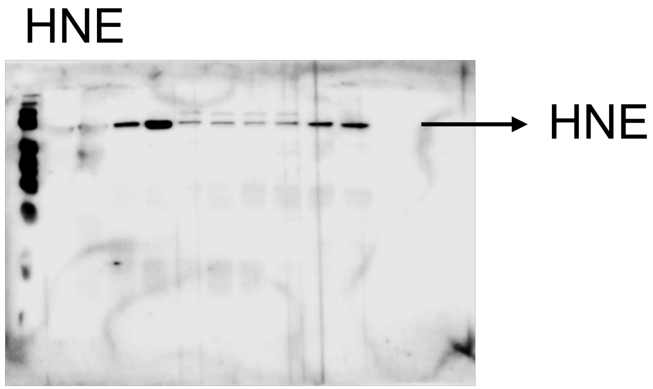

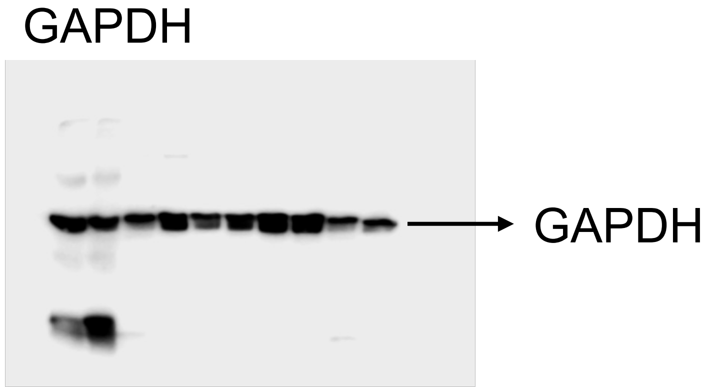

Full unedited blot for Figure 7A

SNCA

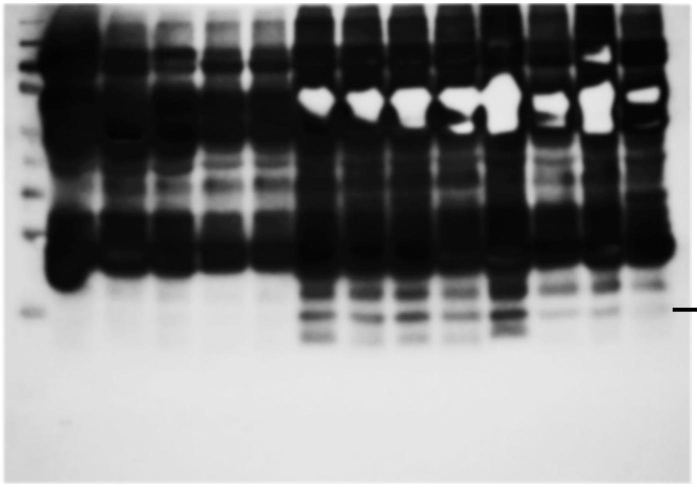

→ SNCA

GAPDH

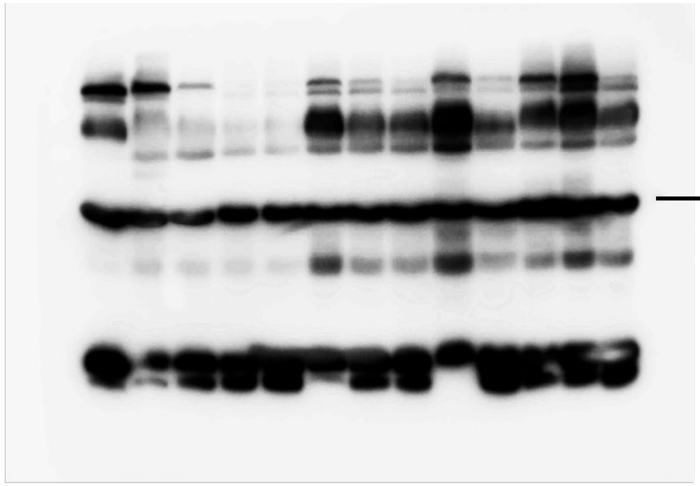

→ GAPDH

Full unedited blot for Figure 7B, Representative

SNCA

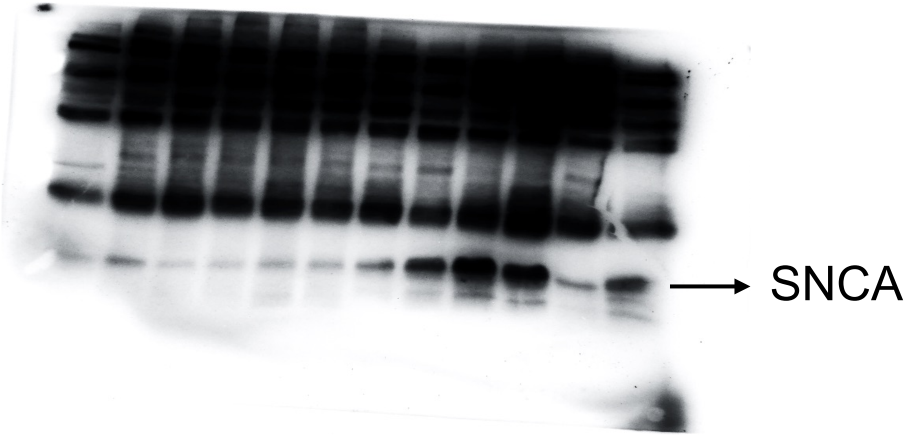

GAPDH

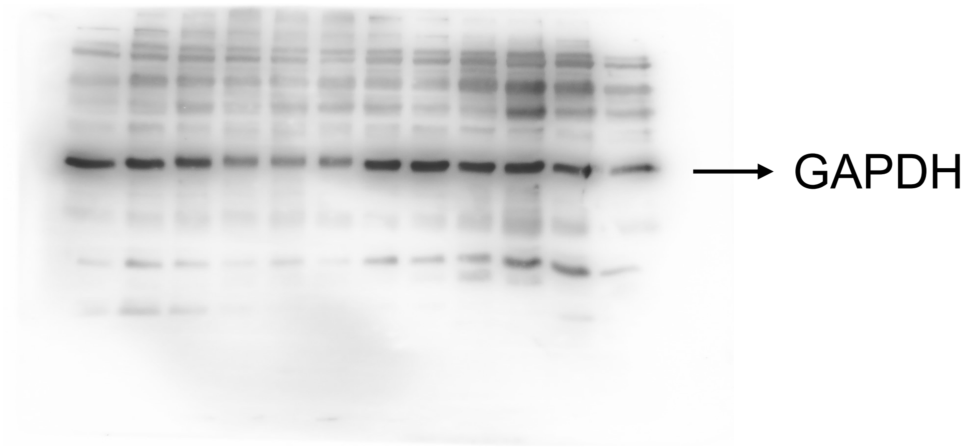

Figure 7B, used in densitometry

SNCA

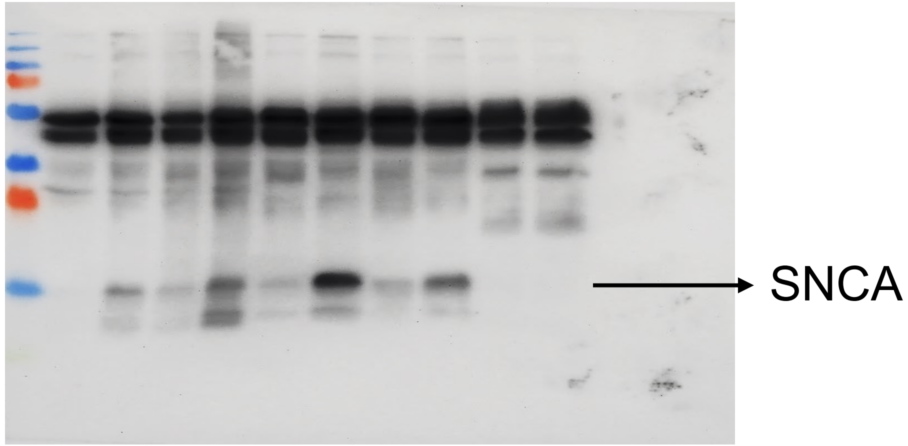

GAPDH

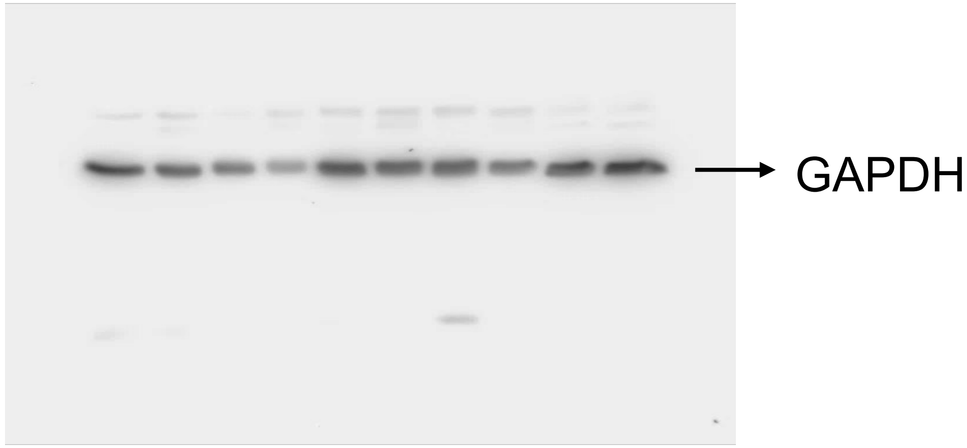

Full unedited blot for Figure 7C

SNCA

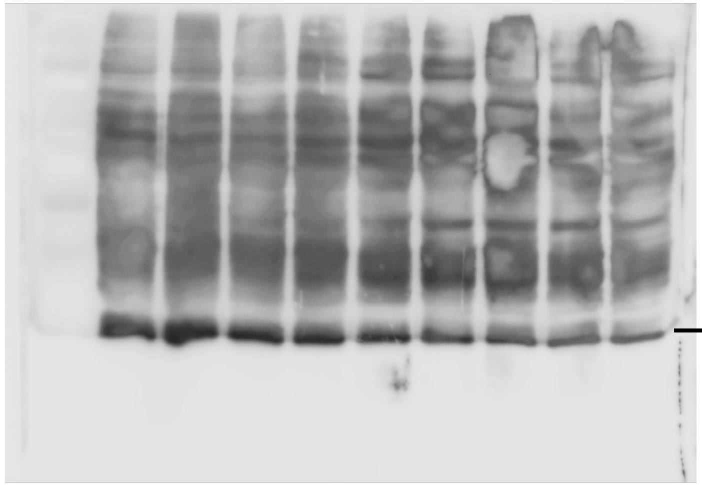

SNCA

GAPDH

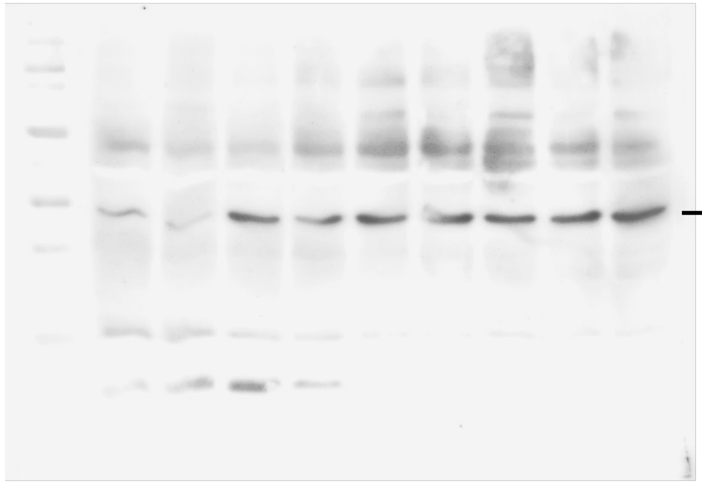

GAPDH

Full unedited blot for Figure 7D

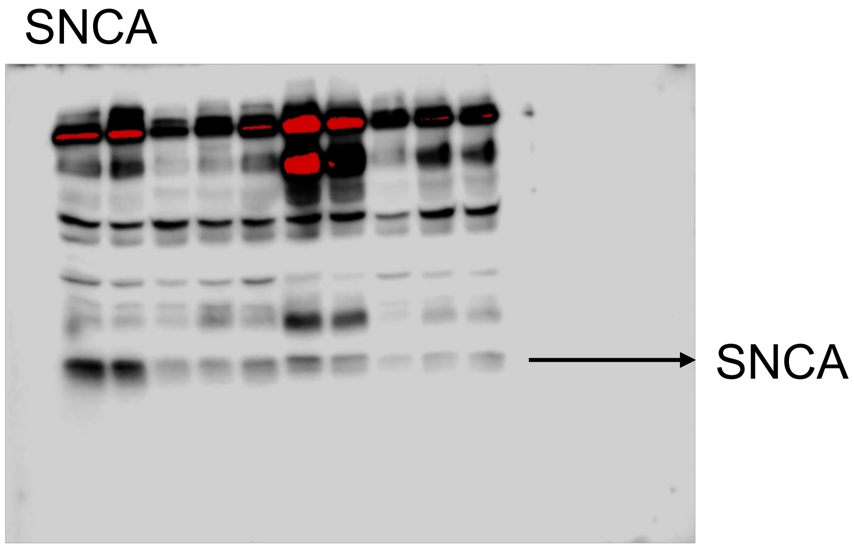

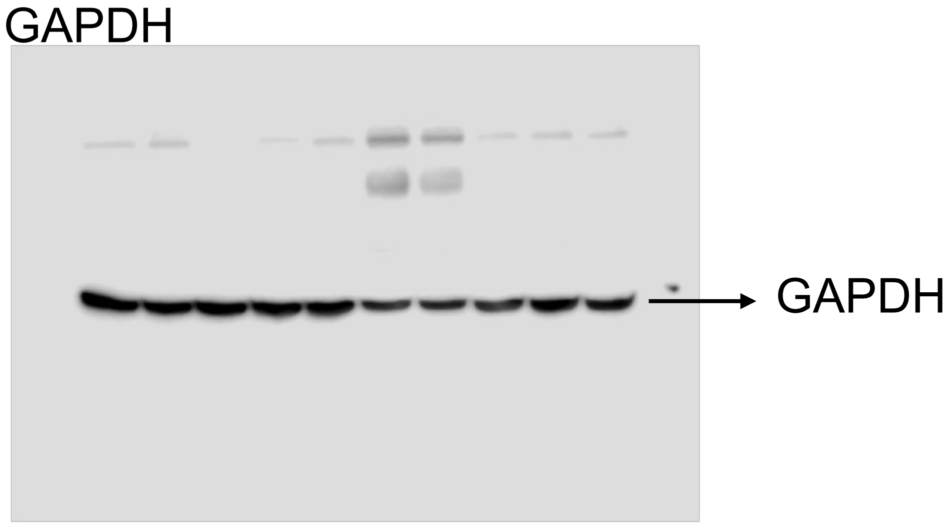

Full unedited blot for Supplemental Figure 7A

HNE

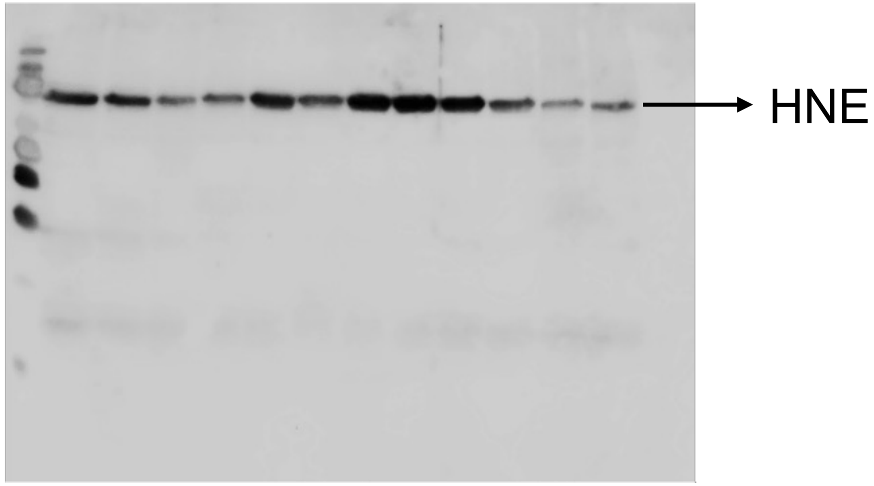

GAPDH

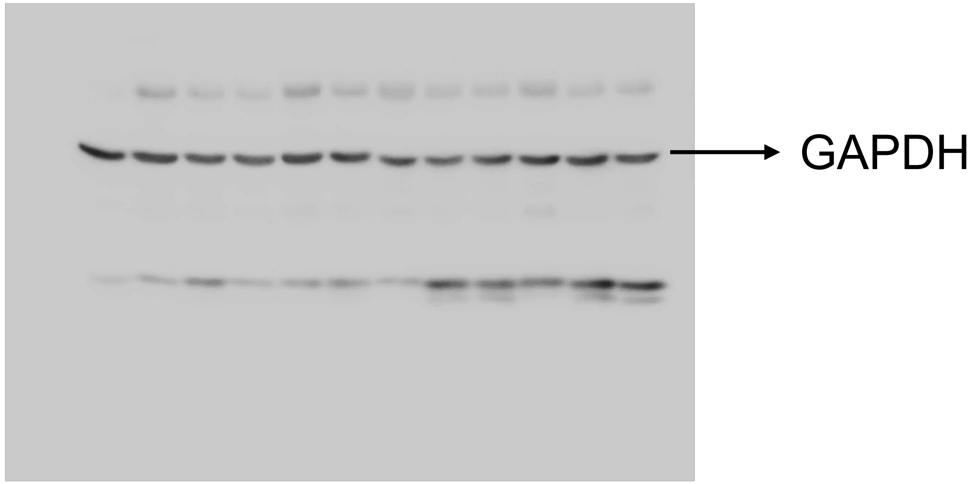

Full unedited blot for Supplemental Figure 7B

ACSL4

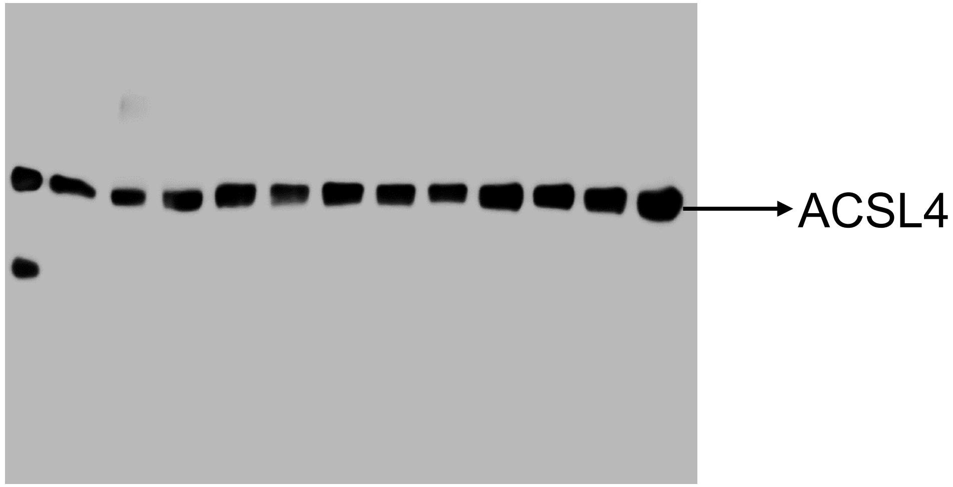

GAPDH

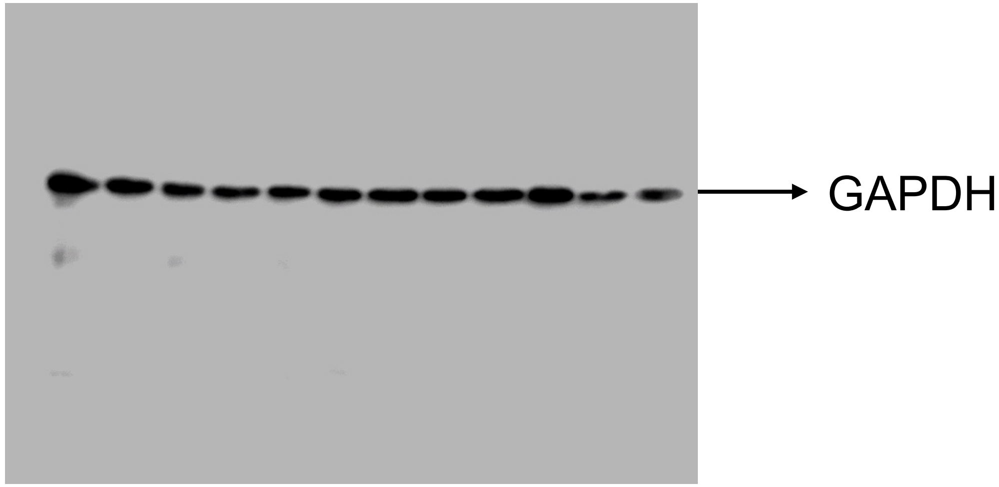

Full unedited blot for Supplemental Figure 8B

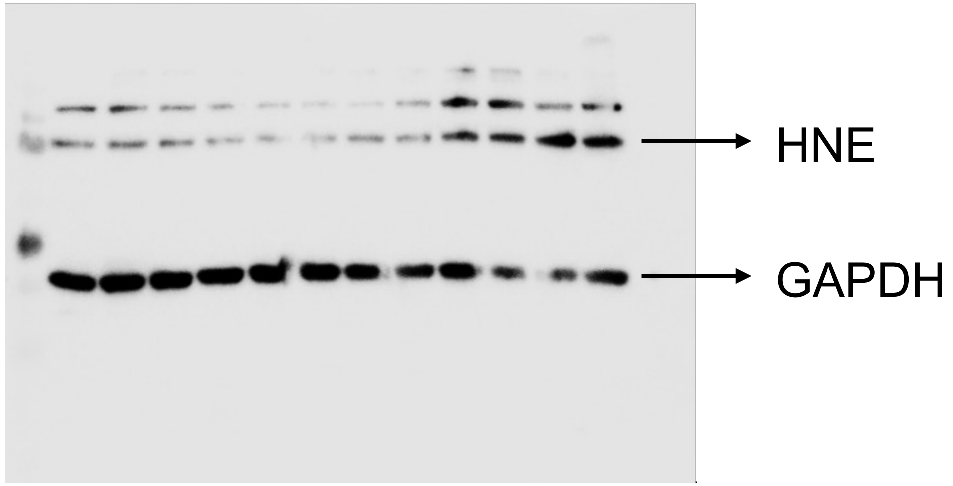

Full unedited blot for Supplemental Figure 8C

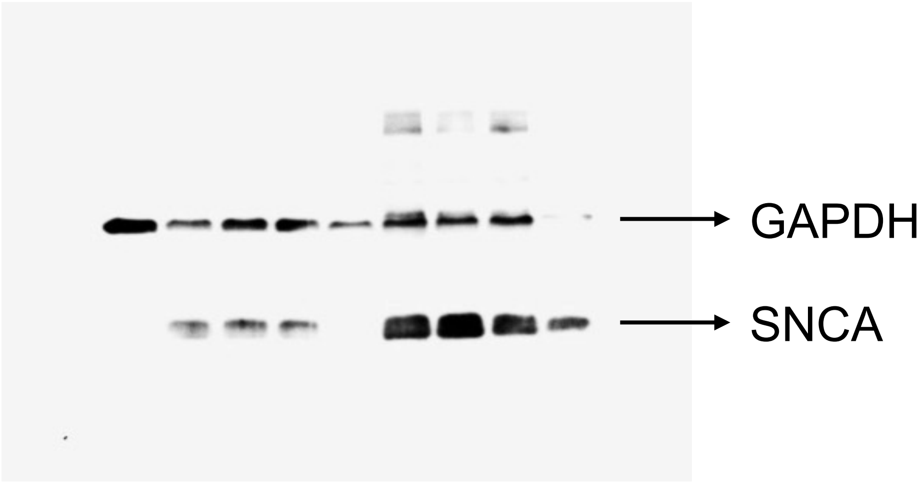

Full unedited blot for Supplemental Figure 9A

FtH

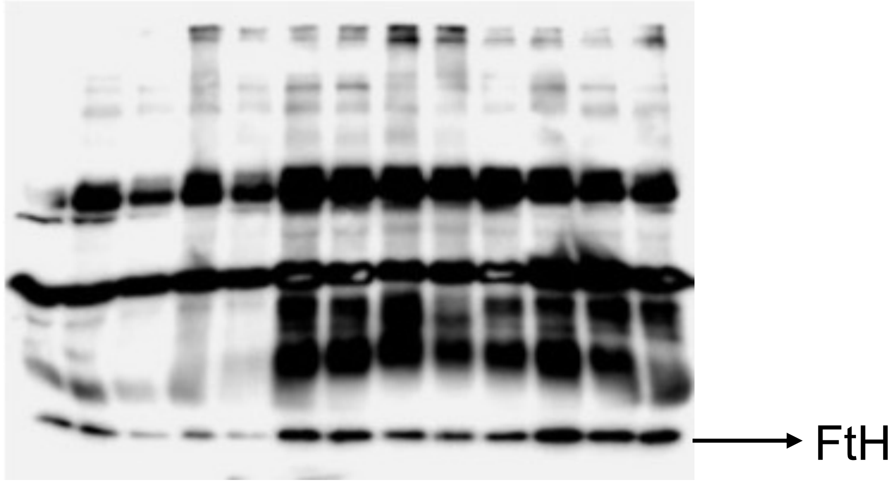

GAPDH

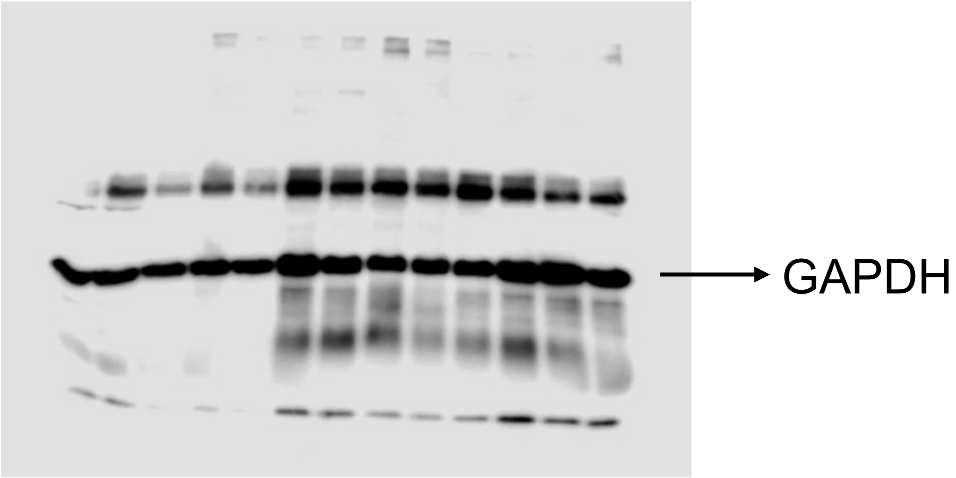

Full unedited blot for Supplemental Figure 9B

FtH

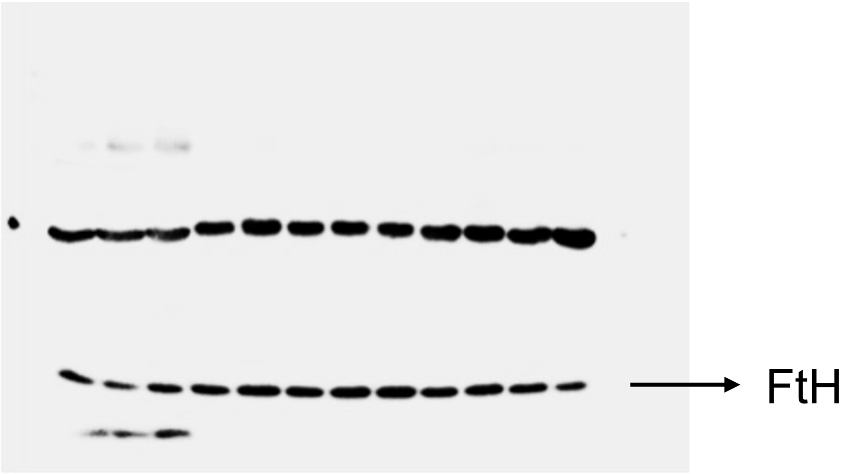

GAPDH

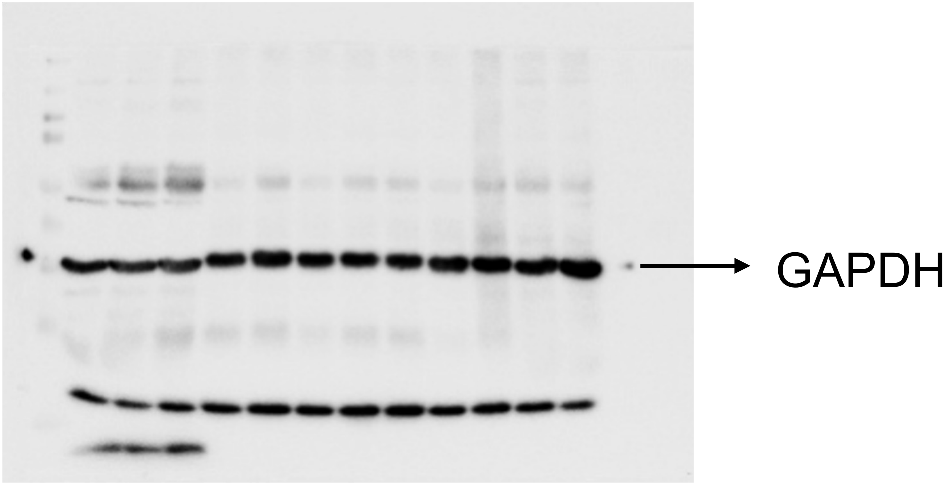

Full unedited blot for Supplemental Figure 9C

FtH

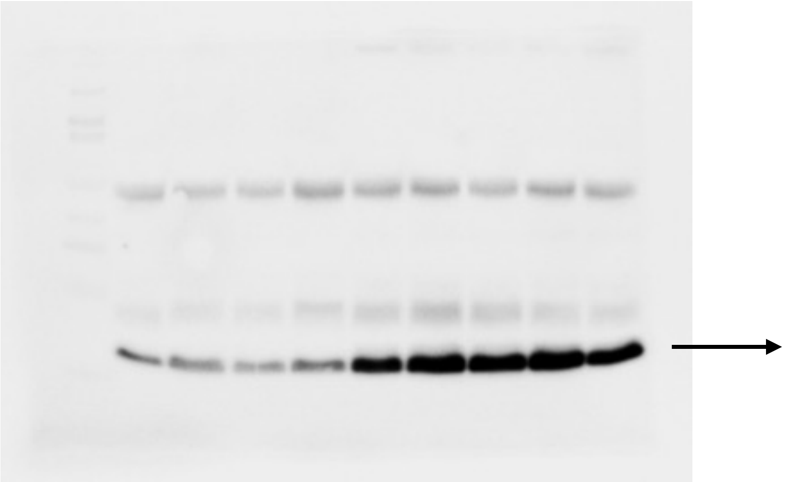

FtH

GAPDH

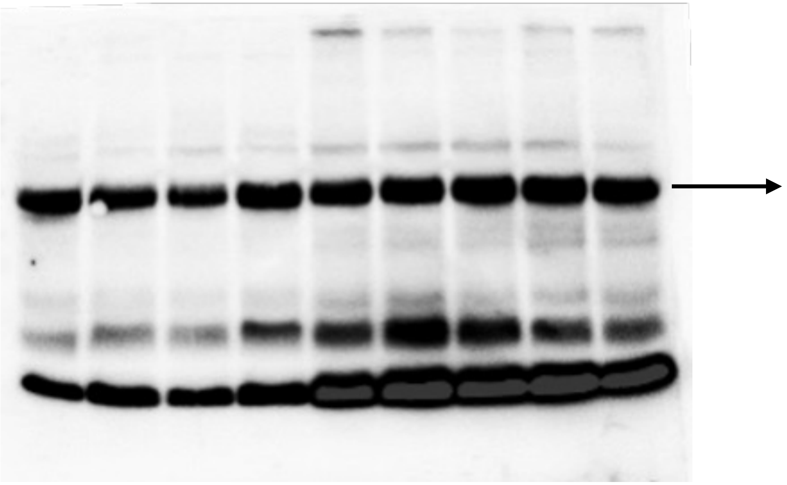

GAPDH

Full unedited blot for Supplemental Figure 9D

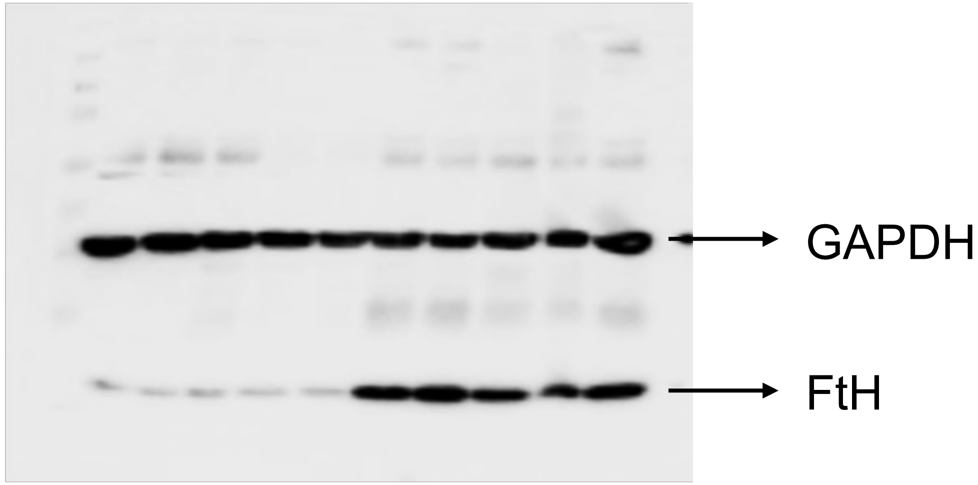

Supplement: Unedited blot and gel images [file jciinsight-11-196521-s095.pdf]
